# Impact of the COVID-19 pandemic on incidence of myocardial infarction, heart failure and stroke, by mental disorder diagnosis, in England, 2019–2023: a cohort study

**DOI:** 10.1136/openhrt-2025-003398

**Published:** 2025-10-22

**Authors:** Kelly Fleetwood, John Nolan, Stewart W Mercer, Sandosh Padmanabhan, Daniel J Smith, Robert Stewart, Caroline A Jackson

**Affiliations:** 1 Usher Institute, University of Edinburgh, Edinburgh, UK; 2 British Heart Foundation Data Science Centre, Health Data Research UK, London, UK; 3 School of Cardiovascular and Metabolic Health, University of Glasgow, Glasgow, UK; 4 Centre for Clinical Brain Sciences, University of Edinburgh, Edinburgh, UK; 5 Department of Psychological Medicine, King’s College London, London, UK; 6 South London and Maudsley NHS Foundation Trust, London, UK

**Keywords:** Myocardial Infarction, HEART FAILURE, STROKE, COVID-19

## Abstract

**ABSTRACT:**

**Background:**

We aimed to estimate mental disorder disparities in cardiovascular disease (CVD) incidence and determine whether these disparities were worsened by the COVID-19 pandemic.

**Methods:**

For each outcome (myocardial infarction (MI), heart failure and stroke), we created a population-based cohort of people without a prior diagnosis of the outcome using linked electronic health records, with follow-up from November 2019 until December 2023. We ascertained pre-existing schizophrenia, bipolar disorder and depression, and each CVD outcome from primary care and hospital admission records and (for CVD outcomes) mortality records. We calculated sex-stratified age-standardised incidence rates by mental disorder diagnosis and used quasi-Poisson modelling to obtain rate ratios (RRs) of CVD among people with each of schizophrenia, bipolar disorder or depression versus those without any of these disorders, adjusting for sociodemographic factors and time period. We investigated whether mental disorder disparities changed as a consequence of the COVID-19 pandemic by including an interaction term between mental disorder and time.

**Results:**

During follow-up, 383 365 people had incident MI, 868 590 had incident heart failure and 455 300 had incident stroke. Age-standardised incidence of each CVD outcome decreased markedly between February and April 2020, with incidence levels returning to, but not exceeding, prepandemic levels in subsequent years. Mental disorder was associated with a higher incidence of each CVD outcome, with RRs ranging from 1.31 (95% CI 1.25 to 1.38) to 2.15 (95% CI 2.05 to 2.24). There was generally no evidence of interaction between mental disorder and time, with mental disorder disparities in CVD incidence stable over time.

**Conclusion:**

We found no clear evidence that the mental disorder disparities in CVD incidence widened during the acute period of the pandemic or during the subsequent years. Continued monitoring of the CVD burden in the general population and among marginalised groups is critical to identifying longer-term impacts on CVD and worsening disparities.

**WHAT IS ALREADY KNOWN ON THIS TOPIC**

**WHAT THIS STUDY ADDS**

- Using contemporary data, this study found that mental disorder disparities in CVD incidence persist.
- There was no evidence of a widening of disparities during or after the pandemic.

**HOW THIS STUDY MIGHT AFFECT RESEARCH, PRACTICE OR POLICY**

- There is an urgent need to effectively address the entrenched mental disorder disparities in CVD incidence.
- Given reduced CVD presentation to both primary and secondary care during the pandemic, continued CVD monitoring, including within marginalised groups such as those with mental disorder, is critical to identifying longer-term impacts on CVD and worsening disparities.

## Introduction

 Mental disorder, irrespective of severity, is associated with increased mortality^1–3^ and reduced life expectancy.^2 4–6^ An excess risk of cardiovascular disease (CVD) is a major contributor to this premature mortality.^2^ Data from Denmark and Scotland revealed large and unchanging disparities in CVD incidence by mental disorder status in the decades up to 2015.^2 7 8^ To our knowledge, these disparities in CVD incidence have not been re-examined in the past 10 years in a universal healthcare setting. Since 2015, the COVID-19 pandemic and the response to it have raised concerns of widening health inequalities in general. With respect to mental health, people with a mental disorder have a greater risk of poor outcomes from COVID-19,^9^ and the pandemic is reported to have exacerbated existing mental disorder disparities in all-cause mortality.^10^ However, the impact of healthcare disruption during the pandemic on mental health disparities in CVD has not yet been examined.

Studies from various settings have consistently demonstrated that, in the general population, there were marked reductions in secondary cardiovascular care activity, including emergency hospital admissions for CVD treatment and procedures during the acute period of the COVID-19 pandemic.^11–16^ Studies outside the UK have reported greater reductions in hospital admissions, including for CVD, among people with schizophrenia versus no mental disorder during 2020.^17^ Furthermore, the early phase of the pandemic resulted in fewer primary care consultations for cardiovascular conditions^18^ and a deficit of diagnoses of long-term conditions, including various cardiovascular conditions, in the general population.^18–20^ Moreover, incident cardiovascular medication prescribing decreased during the first year of the pandemic, raising concerns of increased CVD incidence in the ensuing years, due to underascertainment and undertreatment of cardiovascular risk factors.^21^ We might expect marginalised groups, such as those with mental disorders, to have been disproportionately affected by this health service disruption, and any rebound in CVD occurrence to be greater in this subpopulation. In this study, we aimed to quantify, using contemporary population-based data, mental disorder disparities in myocardial infarction (MI), heart failure and stroke incidence in England and determine whether the COVID-19 pandemic exacerbated these disparities.

## Methods

We have reported this study in accordance with the Reporting of Studies Conducted using Observational Routinely-Collected Health Data statement.^22^

### Setting and data sources

We accessed national electronic health data from England, in National Health Service (NHS) England’s Secure Data Environment (SDE), made available via the British Heart Foundation Data Science Centre’s CVD-COVID-UK/COVID-IMPACT Consortium.^23^ We used the following data sets: General Practice Extraction Service Data for Pandemic Planning and Research (GDPPR), which includes primary care records from individuals in England with active, current registrations at participating practices and deceased patients with a date of death on or after 1 November 2019; Hospital Episode Statistics (HES), which include all inpatient admissions including day cases, and Civil Registration data (for registered deaths in England). Primary care records include the entire medical history for participants, while HES data were included from 1997 to 2023 (inclusive) and death data from November 2019 to December 2023 (inclusive).

### Study design and study population

We conducted three cohort studies (to analyse each CVD outcome separately) with follow-up from 1 November 2019 until 31 December 2023, including people registered in the GDPPR data set. For each cohort, we excluded people with a prior history of each CVD condition accordingly, as well as people whose date of death was recorded as being prior to the date of the CVD event. When estimating age-standardised incidence for the whole cohort, to allow comparability with previously published data on CVD incidence in the UK, we did not impose any age criteria and so included everyone alive on 1 November 2019 or born during follow-up. For analyses of mental disorder and CVD incidence, we included all adults aged at least 40 years on 1 November 2019 or who turned 40 years during follow-up and excluded people aged over 100 years on 1 November 2019.

### Mental disorder

We ascertained schizophrenia, bipolar disorder and depression from the HES and GDPPR data sets. For the HES data set, we used the following International Classification of Diseases, 10th Revision (ICD-10) codes in primary or secondary diagnosis fields: schizophrenia: F20, F25; bipolar disorder: F30, F31; depression: F32, F33. For the GDPPR data set, we identified each disorder using SNOMED codelists (online supplemental text S1). We defined mutually exclusive groups of people with schizophrenia, bipolar disorder or depression using a severity hierarchy, with schizophrenia considered the most severe, followed by bipolar disorder and depression. Individuals entered the exposed group from the date of first record of the most severe of the three disorders.

### Cardiovascular disease

We defined incident MI, heart failure and stroke events as those that occurred in individuals who had no previous record for the event. We identified events occurring between 1 November 2019 and 31 December 2023 from GDPPR diagnoses, primary and secondary diagnosis fields in HES records, and underlying and secondary causes of death from death register data. For the GDPPR data set, we identified events using SNOMED codelists (online supplemental text S1). We identified events from the HES data set and death records using ICD-10 codes as follows: MI (I21, I22); heart failure (I50, I11.0, I13.0, I13.2); and stroke (I60, I61, I63, I64).

### Covariates

For each person in the cohorts, we ascertained age, sex, area-level deprivation (based on quintiles of the Index of Multiple Deprivation, a small-area measure of deprivation, https://www.gov.uk/government/statistics/english-indices-of-deprivation-2019) and ethnicity from the CVD-COVID-UK/COVID-IMPACT Consortium key patient characteristics table (https://bhfdsc.github.io/documentation/curated_assets/kpcs).

### Data preparation

For each CVD outcome and month, we identified people aged at least 40 years by the end of the month, excluding people who died before the start of the month, or with a previous record of the outcome. We calculated the person-years contributed by each person with start of follow-up defined as the latest of first day of the month or date of fortieth birthday, and end of follow-up defined as the earliest of outcome event, death or last day of the month. We summarised the person-years by: age on the last day of the month; sex; deprivation quintile; ethnicity; and mental disorder group. If a person had their first admission for their most severe mental disorder within the month, we allocated the person-years prior to this admission to the non-exposed group and the person-years from this admission to the appropriate mental disorder group. We counted the number of outcome events in the month by age on the last day of the month, sex, deprivation, ethnicity and mental disorder group.

### Statistical analyses

We conducted separate analyses for each of MI, heart failure and stroke. For each outcome, we summarised the sociodemographic characteristics of people with an event by mental disorder diagnosis. In accordance with statistical disclosure control rules, counts are rounded to the nearest 5 and counts less than 10 are suppressed. For each outcome, we also calculated age-standardised incidence rates, per 1000 person-years, using the 2013 European Standard Population (http://www.isdscotland.org/Products-and-Services/GPD-Support/Population/Standard-Populations/).

We applied quasi-Poisson models overall and to sex-stratified cohorts to model the count of each CVD outcome in those aged between 40 years and 100 years by mental disorder, adjusting for age (using both linear and quadratic terms for scaled age), sex (in the overall model), time period and area-level deprivation. We categorised the time period into 4-month intervals because we wanted to capture fluctuations in incidence throughout our study period without making prior assumptions that specific periods (eg, lockdown periods) would have higher or lower incidence rates. In addition to lockdown and relaxation periods, CVD incidence throughout our study period may have been affected by the prevalence of the virus and the roll-out of vaccinations. Moreover, reduced primary and secondary cardiovascular care activity early in the pandemic may have influenced incidence later in our study period. The quasi-Poisson models included log person-years as an offset term. We used the models to estimate rate ratios (RRs) of each outcome for people with each of schizophrenia, bipolar disorder or depression versus those without any of these disorders.

To examine the role of the COVID-19 pandemic on the associations between mental disorder and each outcome, we included interactions between time period and mental disorder, in both the overall and sex-stratified models. As above, we categorised time into 4-month intervals to capture changes in disparities throughout our study period without making prior assumptions that specific periods, such as lockdown periods, would have widened disparities. We fitted the models with and without the interaction and compared their fit using a likelihood ratio test (F-test). If adding the interaction made a statistically significant improvement to the fit of the model, we also tested for differences between the RRs in the prepandemic period (November 2019 to February 2020) and each of the subsequent periods. For all of the models including an interaction, we estimated the RRs for each 4-month interval for people with each mental disorder versus those without any of the disorders.

There were some missing data for deprivation (<1%) and ethnicity (<1%), with missingness of deprivation not varying by mental disorder or outcome status. The pattern of ethnicity missingness was similar in those with schizophrenia, bipolar disorder or depression irrespective of whether an outcome event happened, whereas ethnicity missingness in people without schizophrenia, bipolar disorder or depression was much greater in those not experiencing the outcome compared with those with the outcome (online supplemental table 1). We therefore performed our analyses on the cohorts with complete data on deprivation and conducted a sensitivity analysis where we added ethnicity to the overall model, combining men and women.

The prespecified protocol, codelists and analysis code are available on GitHub (https://github.com/BHFDSC/CCU046_03).

### Patient and public involvement

This project was an extension to another study examining mental disorder and acute care for MI, via the CVD-COVID-UK/COVID-IMPACT Consortium, for which we have an advisory group including lay members and people with lived experience of mental disorders. Details of the current project were shared with the advisory group and ongoing discussions of findings will shape future planned non-academic dissemination activities.

## Results

**Figure 1:**
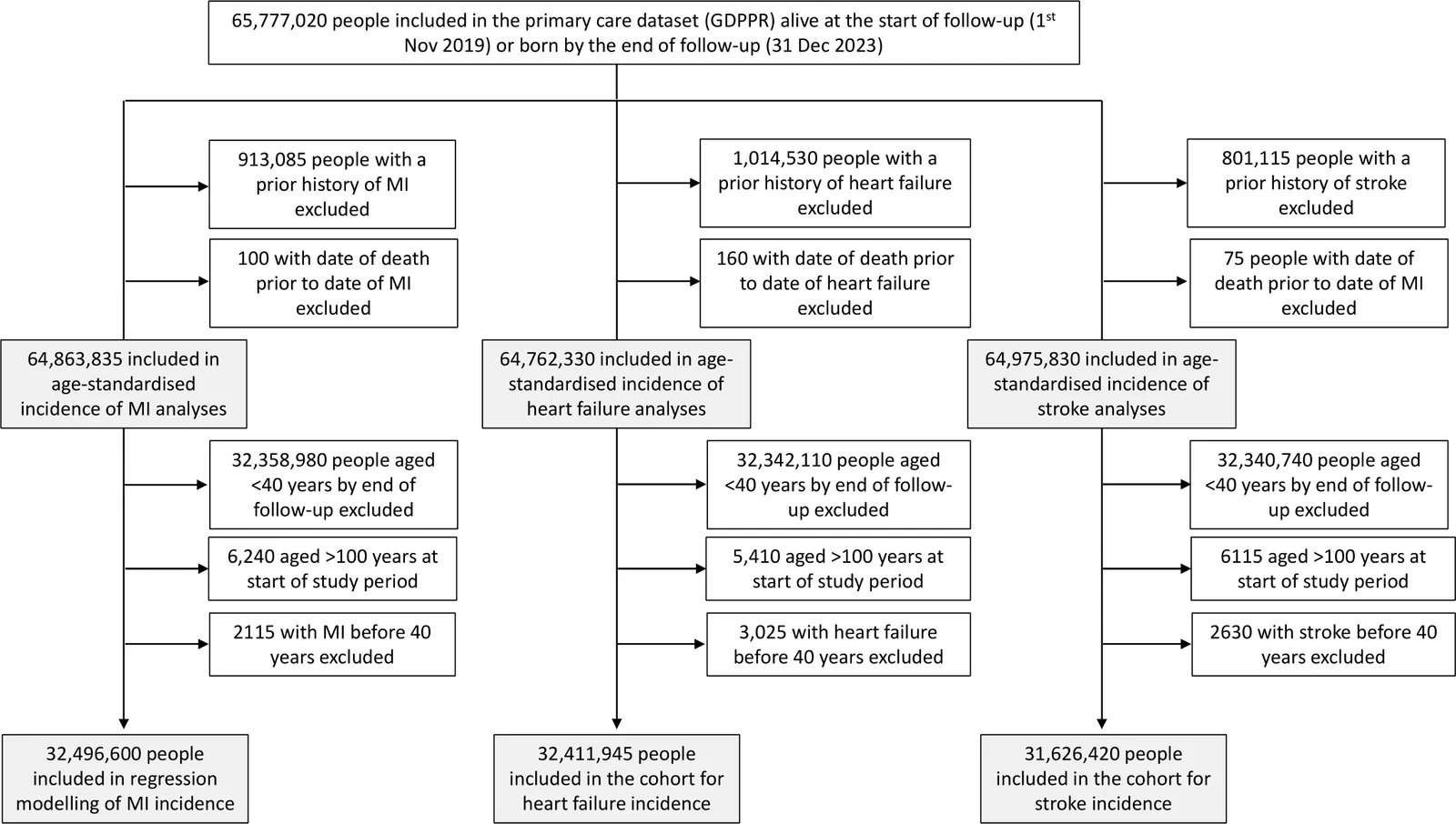
Flow diagram of study populations. In accordance with NHSE’s SDE statistical disclosure control rules, counts are rounded to the nearest 5. GDPPR, General Practice Extraction Service Data for Pandemic Planning and Research; MI, myocardial infarction; NHSE, NHS England; SDE, Secure Data Environment.

We identified 65 777 020 people from the GDPPR data set, alive on 1 November 2019 or born between then and the end of the follow-up period. For each CVD outcome, after applying exclusion criteria, we included approximately 65 million people in the calculation of age-standardised incidence rates. For the analyses of CVD incidence by mental disorder (among people aged 40–100 years) we included approximately 32 million people in each of the MI, heart failure and stroke cohorts (figure 1).

**Figure 2:**
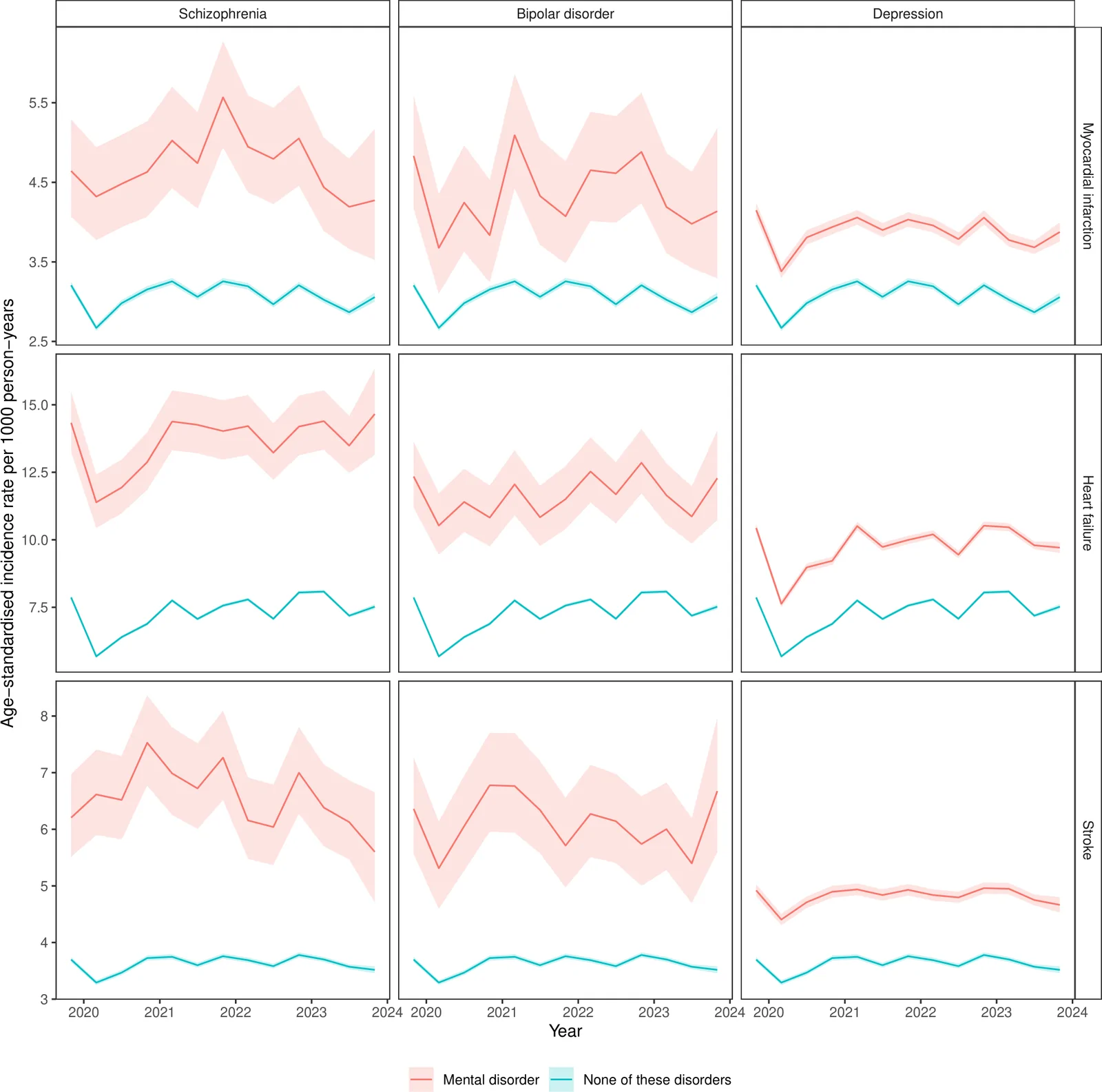
Age-standardised incidence rate for myocardial infarction, heart failure and stroke, by mental disorder diagnosis, in England, November 2019 to December 2023.

In the cohorts without age restriction, age-adjusted incidence of each of MI, heart failure and stroke decreased markedly between February and April 2020, dropping to levels not observed in subsequent years (online supplemental figure 1). These troughs in incidence during the acute phase of the COVID-19 pandemic were evident in people with each of schizophrenia, bipolar disorder and depression, and in those with none of these disorders (figure 2 and online supplemental tables 2–4), with the exception being stroke incidence, for which we did not observe the same trough for people with schizophrenia. Reductions were particularly marked for heart failure incidence. Changes in patterns of CVD incidence were generally similar when stratified by sex (online supplemental figure 2). By 2021, rates of MI, stroke and heart failure largely recovered to prepandemic levels, in all comparison groups.

Among those aged 40–100 years, 383 365 had an MI, 868 590 were diagnosed with heart failure and 455 300 had a stroke (table 1). The mean age of CVD diagnosis among people with each mental disorder was 3–10 years younger compared with those without these disorders, with the gap varying by mental disorder and CVD condition. The gap was largest for heart failure, with people with schizophrenia on average 10 years younger at diagnosis than those without mental disorder. Approximately a quarter to a third of people with a mental disorder and each CVD were living in the most deprived quintile of deprivation, compared with about a fifth of people in the comparison group. The proportion of people who were black, South Asian or of mixed ethnicity was greater among those with schizophrenia, but lower among those with bipolar disorder and depression compared with the comparison group (table 1). Patterns of characteristics by mental disorder diagnosis were similar for men and women (online supplemental tables 5,6).

**Table 1:** Characteristics of people with each cardiovascular disease event, by mental disorder diagnosis

Characteristic	Myocardial infarction (n=383 365)				Heart failure (n=868 590)				Stroke (n=455 300)			
	Schizophrenia (n=3380)	Bipolar disorder (n=2425)	Depression (n=97 615)	None of these disorders (n=279 945)	Schizophrenia (n=8970)	Bipolar disorder (n=5730)	Depression (n=219 645)	None of these disorders (n=634 245)	Schizophrenia (n=4410)	Bipolar disorder (n=3180)	Depression (n=116 395)	None of these disorders (n=331 315)
Age at CVD event (years), mean (±SD)	65 (±14)	65 (±14)	68 (±14)	71 (±14)	67 (±14)	68 (±15)	72 (±14)	77 (±14)	67 (±14)	66 (±15)	70 (±15)	73 (±16)
Female (%)	1325 (38)	1230 (49)	47 620 (48)	92 265 (32)	4330 (47)	3440 (57)	126 685 (56)	274 140 (42)	2115 (47)	1910 (57)	69 690 (58)	153 390 (44)
IMD (quintile)												
1 (most deprived)	1285 (37)	705 (28)	26 430 (27)	56 525 (20)	3225 (35)	1590 (27)	55 485 (25)	119 210 (18)	1505 (33)	900 (27)	28 540 (24)	62 320 (18)
2	885 (25)	565 (23)	21 285 (21)	56 415 (20)	2435 (26)	1295 (22)	47 015 (21)	125 000 (19)	1150 (25)	700 (21)	25 150 (21)	66 375 (19)
3	570 (16)	460 (18)	19 195 (19)	58 455 (21)	1580 (17)	1165 (19)	43 840 (20)	134 250 (21)	820 (18)	680 (20)	23 955 (20)	71 100 (21)
4	450 (13)	420 (17)	17 465 (18)	57 635 (20)	1205 (13)	1040 (17)	41 140 (18)	136 095 (21)	600 (13)	580 (17)	22 525 (19)	72 660 (21)
5 (least deprived)	280 (8)	335 (13)	14 890 (15)	55 005 (19)	780 (8)	855 (14)	35 715 (16)	131 270 (20)	435 (10)	485 (14)	19 465 (16)	70 425 (20)
Missing	30 (1)	15 (1)	595 (1)	1490 (1)	90 (1.0)	50 (1)	1430 (1)	3740 (1)	40 (1)	20 (1)	740 (1)	2040 (1)
Ethnicity												
White	2825 (81)	2295 (92)	91 785 (92)	245 710 (86)	7695 (83)	5565 (93)	211 095 (94)	587 605 (91)	3670 (81)	3100 (92)	111 785 (93)	303 150 (88)
Black	190 (5)	30 (1)	1230 (1)	5685 (2)	650 (7)	100 (2)	2900 (1)	14 990 (2)	330 (7)	65 (2)	2120 (2)	11 515 (3)
South Asian	305 (9)	110 (4)	4070 (4)	21 035 (7)	530 (6)	180 (3)	5860 (3)	26 415 (4)	305 (7)	105 (3)	3420 (3)	15 745 (5)
Mixed	55 (2)	25 (1)	665 (1)	2105 (1)	165 (2)	50 (1)	1325 (1)	4255 (1)	70 (2)	35 (1)	815 (1)	3010 (1)
Other	115 (3)	45 (2)	1895 (2)	9110 (3)	260 (3)	95 (2)	3085 (1)	13 635 (2)	165 (4)	60 (2)	2015 (2)	9730 (3)
Missing	<10 (<0.3)	<10 (<0.4)	215 (0.2)	1880 (1)	<10 (<0.1)	<10 (<0.2)	360 (0.2)	2660 (0.4)	<10 (<0.2)	<10 (<0.3)	225 (0.2)	1765 (0.5)

CVD, cardiovascular disease; IMD, index of multiple deprivation.

**Figure 3:**
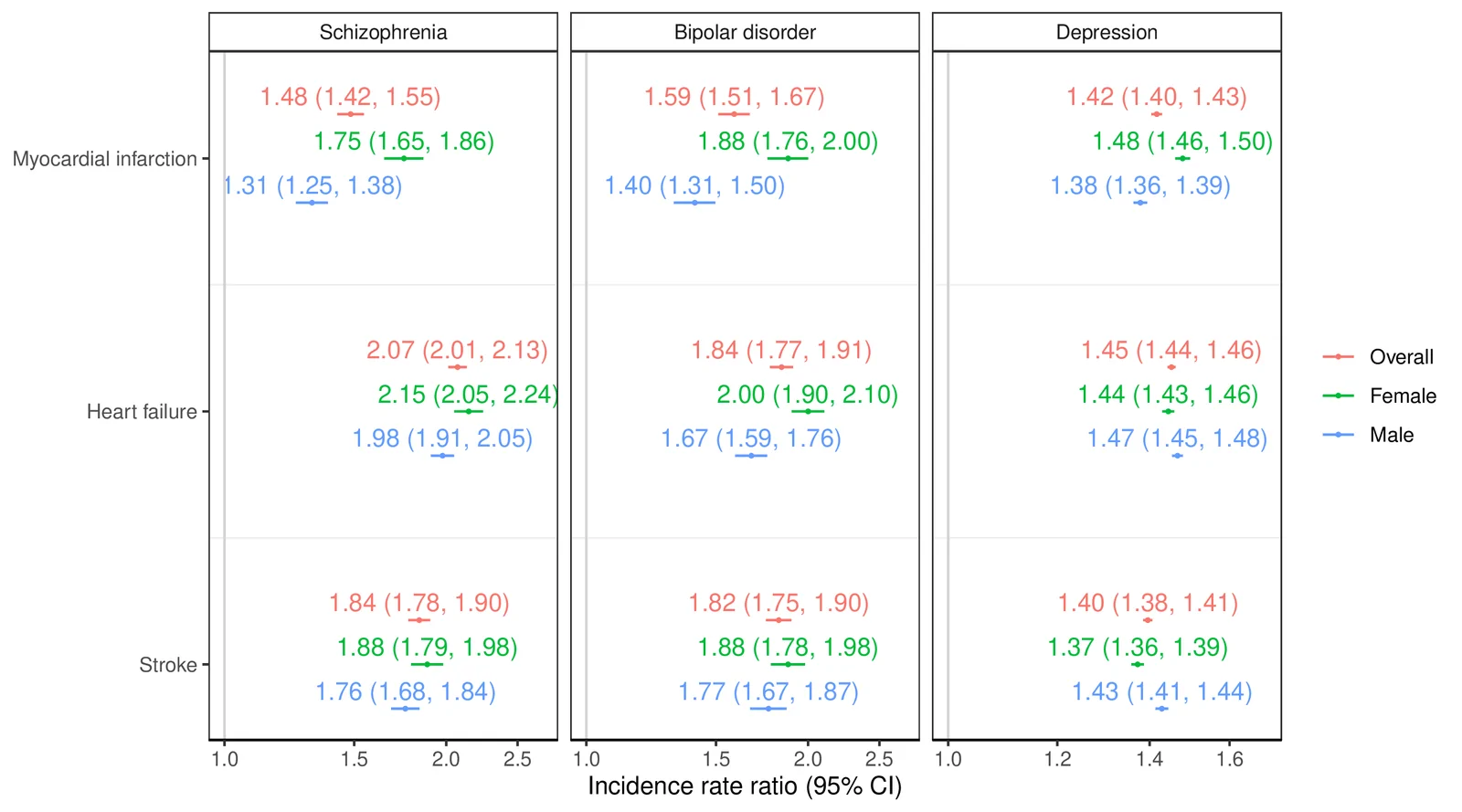
Adjusted rate ratios for the association between each of schizophrenia, bipolar disorder and depression and each of myocardial infarction, heart failure and stroke, for all participants and stratified by sex.* *Models adjusted for age, sex (for the total cohort), deprivation and time period.

In models adjusted for age, sex, area-level deprivation and time period, rates of each CVD were statistically significantly higher in people with each of schizophrenia, bipolar disorder and depression compared with people without any of these mental disorders (figure 3). For the entire period, the rate of MI was 48%, 59% and 42% higher in those with each of schizophrenia (RR 1.48, 95% CI 1.42 to 1.55), bipolar disorder (RR 1.59, 95% CI 1.51 to 1.67) and depression (RR 1.42, 95% CI 1.40 to 1.43), respectively, compared with those without any of these disorders. The rate of heart failure was 107%, 84% and 45% higher in people with each of schizophrenia, bipolar disorder and depression, respectively, compared with people without any of these disorders. Compared with those without schizophrenia, bipolar disorder or depression, women with bipolar disorder had a higher relative rate of heart failure (RR 2.00, 95% CI 1.90 to 2.10) than men (RR 1.67, 95% CI 1.59 to 1.76). The rate of stroke was 84%, 82% and 40% higher in people with each of schizophrenia, bipolar disorder or depression, respectively, compared with those without any of these mental disorders.

Mental disorder disparities in the incidence of each CVD were generally stable over time (online supplemental tables 7–15), with no evidence of statistically significant interaction between mental disorder and time period for most outcomes (online supplemental table 16). For heart failure, although there was a significant interaction between mental disorder and time period overall and for men, on examination of the individual interaction effects, the RRs for mental disorder versus the comparison group only differed from the prepandemic period for depression and only for at most 3 of the 12 subsequent time periods analysed. These widening disparities, although statistically significant, were only very slight (with at most a widening of the gap by a ratio of 1.06) and were not sustained throughout the study period.

The addition of ethnicity did not materially alter the effect estimates (online supplemental tables 16–19, figure 3).

## Discussion

Our analyses revealed that, as expected, CVD incidence was markedly higher in those with versus without schizophrenia, bipolar disorder and depression. However, there was generally no evidence that this disparity worsened during the acute period of the pandemic or during the subsequent years.

The large mental disorder disparities in CVD incidence observed in our study concur with the most recent population-based findings from universal healthcare settings.^7 8^ Within the UK specifically, the effect estimates for MI and stroke obtained in the present study generally align with those of our previous Scottish study.^7^ Slight deviations in the magnitude of association, with the earlier study reporting, for example, larger estimates for depression, are likely explained by differences in methodology, particularly ascertainment of mental disorder via hospital admission records in our previous study compared with primary care and hospital admission records in this study. The present study, leveraging contemporary data, highlights the persistent mental disorder disparities in CVD incidence 10 years on, reinforcing the need for renewed efforts to address these inequities.

Our findings of a decrease in MI, heart failure and stroke incidence between February and April 2020 in the total study population align with previous reports of reduced hospital admissions for these conditions during this time, including in the UK.^11–15^ While the short prepandemic period for which primary care data are currently available in the CVD-COVID-UK/COVID-IMPACT resource limits our comparisons of postpandemic years with the prepandemic period, our overall CVD incidence estimates in the aftermath of the pandemic (2021–2023) are in line with prepandemic estimates from a recent UK study of 20-year time trends of CVD incidence among 20 million people.^24^

To our knowledge, no previous study has compared the impact of the pandemic on CVD occurrence by mental disorder diagnosis. Given the strong links between mental disorder and deprivation, it is interesting that a previous study found no difference in the impact of the pandemic on CVD hospitalisations by deprivation.^25^ Similarly, a previous study found no evidence that the disruption to healthcare delivery has worsened pre-existing social disparities in avoidable hospitalisations.^26^ Interestingly, these findings somewhat contrast with reports of greater healthcare disruption experienced by people in disadvantaged occupational classes.^27^ It is perhaps surprising that our study and others have not revealed a disproportionate health impact among vulnerable population subgroups as a consequence of pandemic-induced healthcare disruption. However, our study findings should be interpreted cautiously and caveated by consideration of the wider context. Competing risk of death during the early phase of the pandemic among those at highest risk of CVD will undoubtedly have contributed to the observed troughs in CVD occurrence observed in our study and others. Moreover, studies have demonstrated that excess mortality during the early phase of the pandemic was higher among those with versus without psychiatric illness,^10^ driven by a higher rate of COVID-19 mortality in this group. While we included deaths where the cause was attributed to MI, heart failure or stroke, sudden deaths of unknown cause, many of which will be due to a major CVD event, were not included. Finally, it may also still be too early to see the emergence of increased rates in CVD incidence, either in the general population or subgroups of the population.

### Strengths and limitations

Our study has a number of strengths. The analysis of a national data set with close to 100% population coverage minimises selection bias. Moreover, we ascertained CVD events and pre-existing mental disorder using both primary and secondary care data, thus reducing underascertainment and misclassification in both exposure and outcome. The use of national data provided a large study population, facilitating analysis of time trends in CVD incidence by sex and by individual mental disorder. Our study provides novel insights since it is the first study, to our knowledge, to examine the indirect impact of the COVID-19 pandemic on CVD incidence in the general population and specifically by mental disorder diagnosis.

Our study has some limitations. Given that the primary care data within the CVD-COVID-UK/COVID-IMPACT resource only included those alive from November 2019 onwards, we were restricted to a relatively short prepandemic period. This limits our ability to compare incidence of CVD in the postpandemic years to the prepandemic period. However, indirect comparisons of CVD incidence patterns from other sources indicate that patterns of CVD incidence from 2021 onwards align with those observed in the prepandemic years.^24^ It was beyond the scope of this study to examine fatal versus non-fatal cardiovascular events or CVD-specific mortality, and so we cannot comment on whether indirect effects of the pandemic have disproportionately affected CVD-specific mortality in people with mental disorders. We did not examine CVD incidence by depression severity since severity is not well recorded in primary care, and we acknowledge that findings may differ by depression severity. Our findings may not be generalisable to other CVD conditions or mental disorders, or to other populations and healthcare settings. Finally, some misclassification of mental disorder status is likely, with ascertainment also likely to have been affected by the COVID-19 pandemic. Underascertainment of mental disorder will have diluted effect estimates and potentially diluted any exacerbations of mental disorder disparities occurring as a result of the pandemic.

Previous findings of deficits in CVD medication prescribing during the first year of the pandemic led to concerns of higher CVD burden in future years.^21^ These predictions are not borne out by our interrogation of CVD incidence in England and our study did not find any evidence that the COVID-19 pandemic disproportionately affected CVD incidence in those with schizophrenia, bipolar disorder or depression versus those without these disorders. Future studies should seek to disentangle the impact of the pandemic on fatal and non-fatal CVD events and whether this varied by mental disorder status. Similarly, future studies should investigate whether there is differential impact on cardiovascular mortality by mental disorder status, in light of reports of ongoing excess cardiovascular mortality in England, as recently as 2023.^28^ Further detailed investigation and monitoring of postpandemic CVD burden is important to establish longer-term effects of the disruption to health services on cardiovascular health and to identify emerging or worsening disparities among marginalised and vulnerable groups such as people with mental disorders.

## Conclusion

Our study found no evidence of a widening of the existing marked mental health disparities in CVD incidence. Continued monitoring of the CVD burden in the general population and among marginalised groups is critical to identifying longer-term impacts on CVD and worsening of disparities among vulnerable subgroups of the population.

## Supplementary material

10.1136/openhrt-2025-003398 online supplemental file 1

## Data Availability

Data may be obtained from a third party and are not publicly available.
