# Supplementary material for: Impact of the COVID-19 pandemic on incidence of myocardial infarction, heart failure and stroke, by mental disorder diagnosis, in England, 2019–2023: a cohort study
Source: Open Heart. 2025 Oct 22;12(2):e003398. doi: 10.1136/openhrt-2025-003398 (PMC12548611; doi:10.1136/openhrt-2025-003398)

**A cohort study of the impact of the COVID-19 pandemic on incidence of myocardial infarction, heart failure and stroke, by mental disorder diagnosis, in England, 2019-2023**

**Supplementary file**

Kelly Fleetwood^1^, John Nolan^2^, Stewart W Mercer^1^, Sandosh Padmanabhan^3^, Daniel J Smith^4^, Robert Stewart^5,6^, Caroline A Jackson^1^, on behalf of the CVD-COVID-UK/COVID-IMPACT Consortium

^1^Usher Institute, University of Edinburgh, Edinburgh, UK

^2^British Heart Foundation Data Science Centre, Health Data Research UK, London, UK

^3^School of Cardiovascular and Metabolic Health, University of Glasgow, Glasgow, UK

^4^Centre for Clinical Brain Sciences, University of Edinburgh, UK

^5^Department of Psychological Medicine, King’s College London, London, UK

^6^South London and Maudsley NHS Foundation Trust, London, UK

**Table of contents**

[Text S1 Development of SNOMED codelists 4](#_Toc209607061)

[Table S1 Missing ethnicity data in the cohorts by CVD event status and mental disorder (adults aged 40 to 100 years) 6](#_Toc209607062)

[Table S2 Age-standardised incidence of myocardial infarction, by mental disorder diagnosis and four-monthly intervals, Nov 2019-Dec 2023 (amongst people aged 40 to 100 years) 7](#_Toc209607063)

[Table S3 Age-standardised incidence of heart failure, by mental disorder diagnosis and four-monthly intervals, Nov 2019-Dec 2023 (amongst people aged 40 to 100 years) 7](#_Toc209607064)

[Table S4 Age-standardised incidence of stroke, by mental disorder diagnosis and four-monthly intervals, Nov 2019-Dec 2023 (amongst people aged 40 to 100 years) 8](#_Toc209607065)

[Table S5 Characteristics of women with each cardiovascular event, by mental disorder diagnosis 9](#_Toc209607066)

[Table S6 Characteristics of men with each cardiovascular event, by mental disorder diagnosis 10](#_Toc209607067)

[Table S7 Adjusted rate ratios for myocardial infarction incidence for people with each of schizophrenia, bipolar disorder and depression versus those without any of these disorders, for each four-month interval, for all participants, based on a quasi-Poisson model including age, sex, deprivation as well as mental disorder diagnosis, time period and their interaction 11](#_Toc209607068)

[Table S8 Adjusted rate ratios for myocardial infarction incidence for people with each of schizophrenia, bipolar disorder and depression versus those without any of these disorders, for each four-month interval, for women, based on a quasi-Poisson model including age and deprivation as well as mental disorder diagnosis, time period and their interaction 11](#_Toc209607069)

[Table S9 Adjusted rate ratios for myocardial infarction incidence for people with each of schizophrenia, bipolar disorder and depression versus those without any of these disorders, for each four-month interval, for men, based on a quasi-Poisson model including age and deprivation as well as mental disorder diagnosis, time period and their interaction 12](#_Toc209607070)

[Table S10 Adjusted rate ratios for heart failure incidence for people with each of schizophrenia, bipolar disorder and depression versus those without any of these disorders, for each four-month interval, for all participants, based on a quasi-Poisson model including age, sex, deprivation as well as mental disorder diagnosis, time period and their interaction 12](#_Toc209607071)

[Table S11 Adjusted rate ratios for heart failure incidence for people with each of schizophrenia, bipolar disorder and depression versus those without any of these disorders, for each four-month interval, for women, based on a quasi-Poisson model including age and deprivation as well as mental disorder diagnosis, time period and their interaction 13](#_Toc209607072)

[Table S12 Adjusted rate ratios for heart failure incidence for people with each of schizophrenia, bipolar disorder and depression versus those without any of these disorders, for each four-month interval, for men, based on a quasi-Poisson model including age and deprivation as well as mental disorder diagnosis, time period and their interaction 13](#_Toc209607073)

[Table S13 Adjusted rate ratios for stroke incidence for people with each of schizophrenia, bipolar disorder and depression versus those without any of these disorders, for each four-month interval, for all participants, based on a quasi-Poisson model including age, sex, deprivation as well as mental disorder diagnosis, time period and their interaction 14](#_Toc209607074)

[Table S14 Adjusted rate ratios for stroke incidence for people with each of schizophrenia, bipolar disorder and depression versus those without any of these disorders, for each four-month interval, for women, based on a quasi-Poisson model including age and deprivation as well as mental disorder diagnosis, time period and their interaction 14](#_Toc209607075)

[Table S15 Adjusted rate ratios for stroke incidence for people with each of schizophrenia, bipolar disorder and depression versus those without any of these disorders, for each four-month interval, for men, based on a quasi-Poisson model including age and deprivation as well as mental disorder diagnosis, time period and their interaction 15](#_Toc209607076)

[Table S16 P-values from the likelihood ratio tests for interaction between mental disorder and time period 15](#_Toc209607077)

[Table S17 Adjusted rate ratios for myocardial infarction incidence for people with each of schizophrenia, bipolar disorder and depression versus those without any of these disorders, for each four-month interval, for all participants, based on a quasi-Poisson model including age, sex, deprivation and ethnicity as well as mental disorder diagnosis, time period and their interaction 16](#_Toc209607078)

[Table S18 Adjusted rate ratios for heart failure incidence for people with each of schizophrenia, bipolar disorder and depression versus those without any of these disorders, for each four-month interval, for all participants, based on a quasi-Poisson model including age, sex, deprivation and ethnicity as well as mental disorder diagnosis, time period and their interaction 16](#_Toc209607079)

[Table S19 Adjusted rate ratios for stroke incidence for people with each of schizophrenia, bipolar disorder and depression versus those without any of these disorders, for each four-month interval, for all participants, based on a quasi-Poisson model including age, sex, deprivation and ethnicity as well as mental disorder diagnosis, time period and their interaction 17](#_Toc209607080)

[Figure S1 Age-standardised incidence of MI, heart failure and stroke in the general population 18](#_Toc209607081)

[Figure S2 Age-standardised incidence of MI, heart failure and stroke, by sex and mental disorder diagnosis (amongst people aged 40 to 100 years) 19](#_Toc209607082)

[Figure S3 Rate ratios for MI, heart failure and stroke incidence in those with each of schizophrenia, bipolar disorder and depression versus those without any of these disorders, with additional adjustment for ethnicity (sensitivity analysis) 20](#_Toc209607083)

# Text S1 Development of SNOMED codelists

The General Practice Extraction Service Data for Pandemic Planning and Research (GDPPR) dataset includes a subset of all SNOMED codes used in the United Kingdom. The subset of codes included is defined by the primary care domain (PCD) reference set.^1^ The PCD reference set is also used by the Quality and Outcomes Framework (QOF) and current and historical versions of the reference set are available from NHS England.^2^ The available SNOMED codes are grouped into code clusters.^1^

**SNOMED codelists for mental disorders**

To develop our SNOMED codelists for mental disorders we extracted codes from all of the clusters related to relevant mental disorder diagnoses: DEPR_COD (depression diagnosis codes), DEPRES_COD (depression resolved codes) and MH_COD (psychosis and schizophrenia and bipolar affective disease codes) from version 47.1 of the QOF cluster list (which was the most recent version at the time of developing the mental disorder codelists).^3^ Additionally, we mapped existing Read v2 and Clinical Terms Version 3 codelists^4^ for schizophrenia, bipolar disorder and depression to SNOMED, and identified codes available within the GDPPR dataset. We collated all SNOMED codes identified from the clusters, and from the mapping process and then identified codes for each of schizophrenia, bipolar disorder or depression.

**SNOMED codelists for cardiovascular disease**

We identified existing SNOMED codelists from the HDR UK phenotype library for each of our cardiovascular disease (CVD) outcomes: myocardial infarction^5^, heart failure^6^ and stroke.^7^ Each of these existing codelists was developed for use with the CVD-COVID-UK resource, however they were based on earlier versions of the PCD reference set. We adapted these codelists for use in our study by reviewing the codes, excluding codes that were not relevant to our study (for example, we wanted to include only current CVD events, so we excluded “history of codes” such as “H/O: Myocardial infarction in last year”), and adding additional relevant codes from the CHD_COD (coronary heart disease codes), HF_COD (heart failure codes), STRK_COD (stroke diagnosis codes), HSTRK_COD (haemorrhagic stroke codes) and OSTR_COD (non-haemorrhagic stroke codes) clusters from version 49.1 of the QOF cluster list (which was the most recent version at the time of developing the CVD codelists).^8^

**Availability of SNOMED codelists**

All of our SNOMED codelists are available via the HDR UK Phenotype Library:

- **Schizophrenia:** <https://phenotypes.healthdatagateway.org/phenotypes/PH1718>
- **Bipolar disorder:** <https://phenotypes.healthdatagateway.org/phenotypes/PH1719>
- **Depression:** <https://phenotypes.healthdatagateway.org/phenotypes/PH1720>
- **Myocardial infarction:** <https://phenotypes.healthdatagateway.org/phenotypes/PH1722>
- **Heart failure:** <https://phenotypes.healthdatagateway.org/phenotypes/PH1721>
- **Stroke:** <https://phenotypes.healthdatagateway.org/phenotypes/PH1723>

**References**

1. NHS Digital. General Practice Extraction Service (GPES) Data for Pandemic Planning and Research (GDPPR): a guide for analysts and users of the data. 2024. Available: <https://digital.nhs.uk/coronavirus/gpes-data-for-pandemic-planning-and-research/guide-for-analysts-and-users-of-the-data> (accessed 13 Jan 2025).
2. NHS Digital. Quality and Outcomes Framework (QOF) business rules. 2024. Available: <https://digital.nhs.uk/data-and-information/data-collections-and-data-sets/data-collections/quality-and-outcomes-framework-qof/business-rules> (accessed 13 Jan 2025)
3. NHS Digital. Quality and Outcomes Framework (QOF) business rules v47.0 2022-2023. 2023. Available: <https://digital.nhs.uk/data-and-information/data-collections-and-data-sets/data-collections/quality-and-outcomes-framework-qof/quality-and-outcome-framework-qof-business-rules/quality-and-outcomes-framework-qof-business-rules-v47.0-2022-2023> (accessed 13 Jan 2025).
4. Prigge R, Fleetwood KJ, Jackson CA, *et al*. Robustly Measuring Multiple Long-Term Health Conditions Using Disparate Linked Datasets in UK Biobank. Available at SSRN: <https://ssrn.com/abstract=4863974>.
5. Wood A, Denholm R, Hollings S, *et al*. PH942 / 2120 - CCU000 Acute Myocardial Infarction (AMI). Phenotype Library. 2022. Available: <http://phenotypes.healthdatagateway.org/phenotypes/PH942/version/2120/detail/>.
6. Knight R, Walker V, Ip S, *et al*. PH968 / 2146 - CCU002_01 Heart failure. Phenotype Library. 2022. Available: <http://phenotypes.healthdatagateway.org/phenotypes/PH968/version/2146/detail/>.
7. Wood A, Denholm R, Hollings S, *et al*. PH948 / 2126 - CCU000 Stroke. Phenotype Library. 2022. Available: <http://phenotypes.healthdatagateway.org/phenotypes/PH948/version/2126/detail/>.
8. NHS Digital. Quality and Outcomes Framework (QOF) business rules v49 2024-2025. 2024. Available: <https://digital.nhs.uk/data-and-information/data-collections-and-data-sets/data-collections/quality-and-outcomes-framework-qof/business-rules/quality-and-outcomes-framework-qof-business-rules-v49-2024-25> (accessed 13 Jan 2025).

# Table S1 Missing ethnicity data in the cohorts by CVD event status and mental disorder (adults aged 40 to 100 years)

| CVD event status | Mental disorder | Myocardial infarction | | Heart failure | | Stroke | |
| --- | --- | --- | --- | --- | --- | --- | --- |
|  |  | N | n (%) | N | n (%) | N | n (%) |
| Event | Schizophrenia | 3380 | <10 (<0.3%) | 8970 | <10 (<0.1%) | 4410 | <10 (<0.2%) |
|  | Bipolar disorder | 2425 | <10 (<0.4%) | 5730 | <10 (<0.2%) | 3180 | <10 (<0.3%) |
|  | Depression | 97 660 | 215 (0.2%) | 219 785 | 355 (0.2%) | 116 475 | 220 (0.2%) |
|  | None of these disorders | 280 155 | 1845 (0.7%) | 635 160 | 2580 (0.4%) | 331 720 | 1690 (0.5%) |
| No event | Schizophrenia | 235 310 | 565 (0.2%) | 225 510 | 560 (0.2%) | 232 045 | 560 (0.2%) |
|  | Bipolar disorder | 184 195 | 395 (0.2%) | 178 510 | 395 (0.2%) | 181 845 | 385 (0.2%) |
|  | Depression | 7 567 755 | 47 880 (0.6%) | 7 401 505 | 47 710 (0.6%) | 7 543 185 | 47 780 (0.6%) |
|  | None of these disorders | 24 125 725 | 732 240 (3.0%) | 23 736 775 | 731 615 (3.1%) | 24 213 560 | 732 350 (3.0%) |

As per NHSE’s SDE statistical disclosure control rules, counts are rounded to the nearest 5 and counts less than 10 are suppressed.

# Table S2 Age-standardised incidence of myocardial infarction, by mental disorder diagnosis and four-monthly intervals, Nov 2019-Dec 2023 (amongst people aged 40 to 100 years)

| **4-month time period** | **Age-standardised incidence, per 100,000 years (95% CI)** | | | |
| --- | --- | --- | --- | --- |
|  | **Schizophrenia** | **Bipolar disorder** | **Depression** | **None of these disorders** |
| Nov 2019 - Feb 2020 | 4.64 (4.07, 5.29) | 4.83 (4.17, 5.59) | 4.15 (4.06, 4.25) | 3.21 (3.16, 3.25) |
| Mar 2020 - Jun 2020 | 4.32 (3.77, 4.94) | 3.68 (3.10, 4.35) | 3.38 (3.30, 3.47) | 2.67 (2.63, 2.71) |
| Jul 2020 - Oct 2020 | 4.48 (3.94, 5.10) | 4.24 (3.63, 4.96) | 3.81 (3.72, 3.90) | 2.98 (2.94, 3.02) |
| Nov 2020 - Feb 2021 | 4.63 (4.07, 5.27) | 3.83 (3.24, 4.53) | 3.94 (3.85, 4.03) | 3.15 (3.11, 3.20) |
| Mar 2021 - Jun 2021 | 5.03 (4.42, 5.70) | 5.09 (4.42, 5.86) | 4.06 (3.97, 4.15) | 3.26 (3.21, 3.30) |
| Jul 2021 - Oct 2021 | 4.74 (4.17, 5.38) | 4.33 (3.71, 5.04) | 3.90 (3.81, 3.99) | 3.06 (3.02, 3.10) |
| Nov 2021 - Feb 2022 | 5.57 (4.94, 6.27) | 4.07 (3.48, 4.77) | 4.03 (3.94, 4.12) | 3.26 (3.21, 3.30) |
| Mar 2022 - Jun 2022 | 4.95 (4.38, 5.59) | 4.65 (4.01, 5.39) | 3.96 (3.87, 4.05) | 3.19 (3.15, 3.23) |
| Jul 2022 - Oct 2022 | 4.79 (4.23, 5.44) | 4.61 (3.99, 5.33) | 3.79 (3.70, 3.87) | 2.97 (2.93, 3.01) |
| Nov 2022 - Feb 2023 | 5.05 (4.46, 5.72) | 4.88 (4.24, 5.62) | 4.06 (3.97, 4.15) | 3.21 (3.17, 3.25) |
| Mar 2023 - Jun 2023 | 4.44 (3.89, 5.06) | 4.19 (3.61, 4.87) | 3.77 (3.69, 3.86) | 3.02 (2.98, 3.06) |
| Jul 2023 - Oct 2023 | 4.19 (3.66, 4.80) | 3.98 (3.42, 4.63) | 3.68 (3.60, 3.77) | 2.87 (2.83, 2.91) |
| Nov 2023 - Dec 2023 | 4.27 (3.53, 5.17) | 4.14 (3.29, 5.18) | 3.88 (3.76, 4.00) | 3.06 (3.00, 3.11) |

CI = confidence interval

# Table S3 Age-standardised incidence of heart failure, by mental disorder diagnosis and four-monthly intervals, Nov 2019-Dec 2023 (amongst people aged 40 to 100 years)

| **4-month time period** | **Age-standardised incidence, per 100,000 years (95% CI)** | | | |
| --- | --- | --- | --- | --- |
|  | **Schizophrenia** | **Bipolar disorder** | **Depression** | **None of these disorders** |
| Nov 2019 - Feb 2020 | 14.33 (13.26, 15.48) | 12.35 (11.18, 13.62) | 10.44 (10.28, 10.60) | 7.86 (7.80, 7.93) |
| Mar 2020 - Jun 2020 | 11.39 (10.44, 12.42) | 10.53 (9.45, 11.72) | 7.63 (7.50, 7.77) | 5.68 (5.62, 5.74) |
| Jul 2020 - Oct 2020 | 11.93 (10.98, 12.97) | 11.40 (10.29, 12.63) | 8.98 (8.83, 9.12) | 6.39 (6.34, 6.45) |
| Nov 2020 - Feb 2021 | 12.88 (11.85, 13.98) | 10.82 (9.76, 12.01) | 9.22 (9.07, 9.37) | 6.89 (6.82, 6.95) |
| Mar 2021 - Jun 2021 | 14.38 (13.32, 15.53) | 12.05 (10.91, 13.31) | 10.51 (10.35, 10.67) | 7.75 (7.69, 7.82) |
| Jul 2021 - Oct 2021 | 14.26 (13.21, 15.39) | 10.83 (9.77, 12.01) | 9.73 (9.59, 9.88) | 7.07 (7.01, 7.13) |
| Nov 2021 - Feb 2022 | 14.03 (12.97, 15.16) | 11.51 (10.40, 12.73) | 9.99 (9.84, 10.15) | 7.56 (7.50, 7.63) |
| Mar 2022 - Jun 2022 | 14.21 (13.14, 15.36) | 12.53 (11.38, 13.79) | 10.20 (10.05, 10.35) | 7.79 (7.72, 7.86) |
| Jul 2022 - Oct 2022 | 13.23 (12.22, 14.31) | 11.68 (10.61, 12.87) | 9.45 (9.31, 9.59) | 7.08 (7.01, 7.14) |
| Nov 2022 - Feb 2023 | 14.19 (13.13, 15.34) | 12.85 (11.71, 14.11) | 10.52 (10.37, 10.68) | 8.05 (7.98, 8.11) |
| Mar 2023 - Jun 2023 | 14.39 (13.34, 15.53) | 11.65 (10.58, 12.83) | 10.46 (10.31, 10.61) | 8.08 (8.02, 8.15) |
| Jul 2023 - Oct 2023 | 13.49 (12.48, 14.58) | 10.86 (9.86, 11.98) | 9.79 (9.65, 9.94) | 7.19 (7.13, 7.25) |
| Nov 2023 - Dec 2023 | 14.66 (13.14, 16.35) | 12.28 (10.74, 14.05) | 9.71 (9.51, 9.91) | 7.52 (7.43, 7.61) |

# Table S4 Age-standardised incidence of stroke, by mental disorder diagnosis and four-monthly intervals, Nov 2019-Dec 2023 (amongst people aged 40 to 100 years)

| **4-month time period** | **Age-standardised incidence, per 100,000 years (95% CI)** | | | |
| --- | --- | --- | --- | --- |
|  | **Schizophrenia** | **Bipolar disorder** | **Depression** | **None of these disorders** |
| Nov 2019 - Feb 2020 | 6.20 (5.52, 6.97) | 6.36 (5.56, 7.27) | 4.92 (4.82, 5.03) | 3.70 (3.65, 3.74) |
| Mar 2020 - Jun 2020 | 6.61 (5.90, 7.41) | 5.31 (4.60, 6.14) | 4.40 (4.31, 4.50) | 3.29 (3.25, 3.33) |
| Jul 2020 - Oct 2020 | 6.52 (5.82, 7.29) | 6.05 (5.27, 6.95) | 4.71 (4.61, 4.81) | 3.47 (3.43, 3.51) |
| Nov 2020 - Feb 2021 | 7.53 (6.77, 8.36) | 6.78 (5.96, 7.70) | 4.90 (4.79, 5.00) | 3.72 (3.68, 3.77) |
| Mar 2021 - Jun 2021 | 6.99 (6.25, 7.80) | 6.76 (5.94, 7.70) | 4.94 (4.84, 5.04) | 3.75 (3.70, 3.79) |
| Jul 2021 - Oct 2021 | 6.72 (6.01, 7.51) | 6.34 (5.58, 7.21) | 4.84 (4.74, 4.94) | 3.60 (3.56, 3.64) |
| Nov 2021 - Feb 2022 | 7.27 (6.52, 8.10) | 5.71 (4.97, 6.56) | 4.93 (4.83, 5.03) | 3.76 (3.71, 3.80) |
| Mar 2022 - Jun 2022 | 6.16 (5.48, 6.91) | 6.27 (5.51, 7.14) | 4.84 (4.74, 4.94) | 3.69 (3.64, 3.73) |
| Jul 2022 - Oct 2022 | 6.04 (5.37, 6.79) | 6.14 (5.40, 6.98) | 4.80 (4.70, 4.89) | 3.58 (3.54, 3.63) |
| Nov 2022 - Feb 2023 | 7.00 (6.27, 7.80) | 5.74 (5.00, 6.58) | 4.96 (4.86, 5.06) | 3.78 (3.74, 3.82) |
| Mar 2023 - Jun 2023 | 6.38 (5.70, 7.14) | 6.00 (5.28, 6.83) | 4.95 (4.85, 5.05) | 3.70 (3.66, 3.74) |
| Jul 2023 - Oct 2023 | 6.13 (5.47, 6.86) | 5.40 (4.70, 6.20) | 4.75 (4.66, 4.85) | 3.57 (3.53, 3.61) |
| Nov 2023 - Dec 2023 | 5.60 (4.71, 6.66) | 6.68 (5.59, 7.96) | 4.66 (4.53, 4.80) | 3.52 (3.46, 3.58) |

# Table S5 Characteristics of women with each cardiovascular event, by mental disorder diagnosis

| **Characteristic** | **Myocardial infarction** | | | | **Heart failure** | | | | **Stroke** | | | |
| --- | --- | --- | --- | --- | --- | --- | --- | --- | --- | --- | --- | --- |
|  | Schizophrenia  (N=1300) | Bipolar disorder  (N=1190) | Depression  (N=46,780) | None of these disorders  (N=91,005) | Schizophrenia  (N=4240) | Bipolar disorder  (N=3275) | Depression  (N=124,105) | None of these disorders  (N=268,125) | Schizophrenia  (N=2070) | Bipolar disorder  (N=1790) | Depression  (N=67,420) | None of these disorders  (N=147,450) |
| Age at CVD event (years), mean (±SD) | 71 (±12) | 68 (±13) | 72 (±13) | 77 (±13) | 71 (±12) | 70 (±13) | 75 (±12) | 80 (±11) | 72 (±13) | 69 (±14) | 78 (±13) | 76 (±13) |
| IMD (quintile) |  |  |  |  |  |  |  |  |  |  |  |  |
| 1 (most  deprived) | 440 (34) | 345 (29) | 11,995 (26) | 17,905 (20) | 1390 (33) | 880 (27) | 29,870 (24) | 48,770 (18) | 620 (30) | 495 (28) | 15,045 (22) | 25,190 (17) |
| 2 | 315 (24) | 290 (24) | 10,030 (21) | 17,880 (20) | 1075 (25) | 725 (22) | 25,835 (21) | 81,735 (19) | 510 (25) | 365 (20) | 13,910 (21) | 27,925 (19) |
| 3 | 215 (17) | 210 (18) | 9150 (20) | 18,670 (21) | 730 (17) | 630 (19) | 24,335 (20) | 55,205 (21) | 390 (19) | 355 (20) | 13,495 (20) | 30,680 (21) |
| 4 | 195 (15) | 185 (16) | 8220 (18) | 18,335 (20) | 605 (14) | 575 (18) | 22,985 (19) | 56,225 (21) | 300 (15) | 305 (17) | 13,095 (19) | 31,815 (22) |
| 5 (least  deprived) | 125 (10) | 155 (13) | 7090 (15) | 17,675 (19) | 405 (10) | 440 (13) | 20,280 (16) | 54,475 (20) | 230 (11) | 260 (15) | 11,485 (17) | 30,905 (21) |
| Missing | 15 (1) | <10 (<1) | 290 (1) | 545 (1) | 40 (1.0) | 50 (1) | 805 (1) | 1715 (1) | 15 (1) | 10 (1) | 390 (1) | 935 (1) |
| Ethnicity |  |  |  |  |  |  |  |  |  |  |  |  |
| White | 1065 (82) | 1110 (93) | 43,450 (92) | 80,460 (88) | 3505 (83) | 3065 (94) | 117,150 (94) | 244,325 (91) | 1665 (80) | 1670 (93) | 62,980 (93) | 131,555 (89) |
| Black | 75 (6) | 20 (2) | 565 (1) | 1905 (2) | 290 (7) | 60 (2) | 1600 (1) | 6050 (2) | 160 (8) | 30 (2) | 1135 (2) | 4535 (3) |
| South Asian | 105 (8) | 35 (3) | 1705 (4) | 5250 (6) | 265 (6) | 80 (2) | 3000 (2) | 10,105 (4) | 140 (7) | 45 (3) | 1775 (3) | 5850 (4) |
| Mixed | 15 (1) | <10 (<1) | 275 (1) | 585 (1) | 65 (2) | 25 (1) | 645 (1) | 1605 (1) | 20 (1) | 20 (1) | 425 (1) | 1090 (1) |
| Other | 40 (3) | 15 (1) | 715 (2) | 2330 (3) | 115 (3) | 45 (1) | 1530 (1) | 4975 (2) | 80 (4) | 25 (1) | 985 (2) | 3680 (3) |
| Missing | <10 (<1) | <10 (<0.4) | 70 (0.1) | 480 (1) | <10 (<0.2) | <10 (<0.3) | 180 (0.1) | 1060 (0.4) | <10 (<0.5) | <10 (<0.6) | 115 (0.2) | 740 (1) |

CVD = cardiovascular disease; IMD = index of multiple deprivation; SD = standard deviation

# Table S6 Characteristics of men with each cardiovascular event, by mental disorder diagnosis

| **Characteristic** | **Myocardial infarction** | | | | **Heart failure** | | | | **Stroke** | | | |
| --- | --- | --- | --- | --- | --- | --- | --- | --- | --- | --- | --- | --- |
|  | Schizophrenia  (N=2075) | Bipolar disorder  (N=1235) | Depression  (N=50,835) | None of these disorders  (N=188,940) | Schizophrenia  (N=4730) | Bipolar disorder  (N=2455) | Depression  (N=95,540) | None of these disorders (N=366,120) | Schizophrenia  (N=2335) | Bipolar disorder  (N=1390) | Depression  (N=48,975) | None of these disorders (N=183,865) |
| Age at CVD event (years), mean (±SD) | 63 (±12) | 65 (±12) | 66 (±12) | 69 (±13) | 65 (±12) | 69 (±12) | 71 (±12) | 75 (±12) | 65 (±12) | 67 (±12) | 69 (±13) | 72 (±13) |
| IMD (quintile) |  |  |  |  |  |  |  |  |  |  |  |  |
| 1 (most  deprived) | 795 (38) | 330 (27) | 13,545 (26) | 36,765 (20) | 1710 (36) | 615 (25) | 23,805 (25) | 66,135 (18) | 820 (35) | 345 (25) | 12,155 (25) | 33,535 (18) |
| 2 | 530 (26) | 250 (20) | 10,715 (21) | 37,195 (20) | 1255 (27) | 510 (21) | 20,010 (21) | 69,515 (19) | 595 (26) | 280 (20) | 10,340 (21) | 35,395 (19) |
| 3 | 345 (17) | 240 (19) | 9690 (19) | 38,795 (21) | 795 (17) | 500 (20) | 18,680 (20) | 76,235 (21) | 410 (18) | 290 (21) | 9755 (20) | 37,835 (21) |
| 4 | 240 (12) | 230 (18) | 8990 (18) | 38,545 (20) | 570 (12) | 425 (17) | 17,510 (18) | 77,590 (21) | 290 (12) | 255 (18) | 8865 (18) | 38,590 (21) |
| 5 (least  deprived) | 150 (7) | 175 (14) | 7605 (15) | 36,715 (19) | 355 (8) | 385 (16) | 14,935 (16) | 74,700 (20) | 200 (109 | 210 (15) | 7540 (15) | 37,470 (20) |
| Missing | 15 (1) | 10 (1) | 295 (1) | 920 (1) | 45 (1.0) | 20 (1) | 605 (1) | 1945 (1) | 20 (1) | <10 (<1) | 325 (1) | 1040 (1) |
| Ethnicity |  |  |  |  |  |  |  |  |  |  |  |  |
| White | 1675 (81) | 1115 (90) | 46,415 (91) | 161,675 (86) | 3950 (84) | 2270 (93) | 89,570 (94) | 332,440 (91) | 1895 (81) | 1270 (91) | 45,270 (92) | 210,260 (89) |
| Black | 100 (5) | <10 (<1) | 630 (1) | 3505 (1) | 315 (7) | 30 (1) | 1140 (1) | 7695 (2) | 160 (7) | 25 (2) | 855 (2) | 7005 (3) |
| South Asian | 190 (9) | 70 (6) | 2215 (4) | 14,665 (8) | 235 (5) | 90 (4) | 2645 (3) | 14,595 (4) | 150 (6) | 55 (4) | 1490 (3) | 10,165 (4) |
| Mixed | 40 (2) | 15 (1) | 335 (1) | 1400 (1) | 95 (2) | 20 (1) | 565 (1) | 2150 (1) | 45 (2) | 10 (1) | 325 (1) | 1870 (1) |
| Other | 70 (3) | 20 (2) | 1100 (2) | 6330 (3) | 135 (3) | 45 (2) | 1445 (2) | 7720 (2) | 85 (4) | 25 (2) | 930 (2) | 6200 (3) |
| Missing | <10 (<0.5) | <10 (<0.8) | 145 (0.3) | 1365 (1) | <10 (<0.2) | <10 (<0.4) | 175 (0.2) | 1515 (0.4) | <10 (<0.4) | <10 (<0.7) | 105 (0.2) | 1060 (0.4) |

CVD = cardiovascular disease; IMD = index of multiple deprivation; SD = standard deviation

# Table S7 Adjusted rate ratios for myocardial infarction incidence for people with each of schizophrenia, bipolar disorder and depression versus those without any of these disorders, for each four-month interval, for all participants, based on a quasi-Poisson model including age, sex, deprivation as well as mental disorder diagnosis, time period and their interaction

| Time period | Schizophrenia | Bipolar disorder | Depression |
| --- | --- | --- | --- |
| Nov 2019 - Feb 2020 | 1.37 (1.17, 1.59) | 1.73 (1.46, 2.05) | 1.45 (1.40, 1.49) |
| Mar 2020 - Jun 2020 | 1.56 (1.34, 1.83) | 1.54 (1.27, 1.87) | 1.42 (1.37, 1.47) |
| Jul 2020 - Oct 2020 | 1.52 (1.31, 1.76) | 1.57 (1.31, 1.89) | 1.44 (1.39, 1.48) |
| Nov 2020 - Feb 2021 | 1.46 (1.26, 1.70) | 1.33 (1.10, 1.61) | 1.40 (1.36, 1.45) |
| Mar 2021 - Jun 2021 | 1.45 (1.25, 1.67) | 1.78 (1.52, 2.09) | 1.40 (1.36, 1.45) |
| Jul 2021 - Oct 2021 | 1.51 (1.31, 1.75) | 1.57 (1.32, 1.87) | 1.44 (1.39, 1.48) |
| Nov 2021 - Feb 2022 | 1.64 (1.43, 1.89) | 1.44 (1.20, 1.72) | 1.39 (1.34, 1.43) |
| Mar 2022 - Jun 2022 | 1.56 (1.35, 1.79) | 1.59 (1.34, 1.88) | 1.40 (1.36, 1.44) |
| Jul 2022 - Oct 2022 | 1.56 (1.35, 1.80) | 1.72 (1.46, 2.03) | 1.43 (1.39, 1.48) |
| Nov 2022 - Feb 2023 | 1.51 (1.31, 1.74) | 1.71 (1.46, 2.01) | 1.41 (1.37, 1.46) |
| Mar 2023 - Jun 2023 | 1.38 (1.19, 1.61) | 1.57 (1.32, 1.86) | 1.41 (1.36, 1.45) |
| Jul 2023 - Oct 2023 | 1.37 (1.17, 1.60) | 1.58 (1.33, 1.88) | 1.44 (1.39, 1.48) |
| Nov 2023 - Dec 2023 | 1.31 (1.05, 1.62) | 1.40 (1.09, 1.80) | 1.41 (1.35, 1.47) |

# Table S8 Adjusted rate ratios for myocardial infarction incidence for people with each of schizophrenia, bipolar disorder and depression versus those without any of these disorders, for each four-month interval, for women, based on a quasi-Poisson model including age and deprivation as well as mental disorder diagnosis, time period and their interaction

| Time period | Schizophrenia | Bipolar disorder | Depression |
| --- | --- | --- | --- |
| Nov 2019 - Feb 2020 | 1.66 (1.34, 2.05) | 1.92 (1.53, 2.39) | 1.49 (1.42, 1.55) |
| Mar 2020 - Jun 2020 | 1.96 (1.57, 2.45) | 1.70 (1.31, 2.22) | 1.52 (1.45, 1.60) |
| Jul 2020 - Oct 2020 | 1.62 (1.29, 2.03) | 1.74 (1.37, 2.20) | 1.48 (1.41, 1.54) |
| Nov 2020 - Feb 2021 | 1.49 (1.18, 1.89) | 1.55 (1.21, 1.99) | 1.46 (1.40, 1.53) |
| Mar 2021 - Jun 2021 | 1.78 (1.44, 2.20) | 2.02 (1.63, 2.50) | 1.46 (1.40, 1.53) |
| Jul 2021 - Oct 2021 | 1.83 (1.49, 2.26) | 1.78 (1.41, 2.24) | 1.46 (1.40, 1.52) |
| Nov 2021 - Feb 2022 | 2.12 (1.75, 2.57) | 1.74 (1.39, 2.19) | 1.42 (1.36, 1.48) |
| Mar 2022 - Jun 2022 | 1.77 (1.43, 2.19) | 1.90 (1.52, 2.37) | 1.47 (1.41, 1.53) |
| Jul 2022 - Oct 2022 | 1.88 (1.53, 2.32) | 2.02 (1.63, 2.50) | 1.47 (1.41, 1.53) |
| Nov 2022 - Feb 2023 | 1.71 (1.38, 2.12) | 2.19 (1.79, 2.68) | 1.52 (1.46, 1.59) |
| Mar 2023 - Jun 2023 | 1.77 (1.43, 2.21) | 1.98 (1.59, 2.46) | 1.50 (1.43, 1.57) |
| Jul 2023 - Oct 2023 | 1.62 (1.29, 2.04) | 2.02 (1.63, 2.50) | 1.52 (1.45, 1.59) |
| Nov 2023 - Dec 2023 | 1.42 (1.02, 1.98) | 1.74 (1.27, 2.38) | 1.48 (1.39, 1.57) |

# Table S9 Adjusted rate ratios for myocardial infarction incidence for people with each of schizophrenia, bipolar disorder and depression versus those without any of these disorders, for each four-month interval, for men, based on a quasi-Poisson model including age and deprivation as well as mental disorder diagnosis, time period and their interaction

| Time period | Schizophrenia | Bipolar disorder | Depression |
| --- | --- | --- | --- |
| Nov 2019 - Feb 2020 | 1.18 (0.98, 1.43) | 1.60 (1.29, 2.00) | 1.41 (1.35, 1.47) |
| Mar 2020 - Jun 2020 | 1.34 (1.11, 1.62) | 1.45 (1.14, 1.86) | 1.37 (1.31, 1.43) |
| Jul 2020 - Oct 2020 | 1.42 (1.19, 1.69) | 1.47 (1.16, 1.85) | 1.41 (1.35, 1.47) |
| Nov 2020 - Feb 2021 | 1.41 (1.18, 1.67) | 1.19 (0.93, 1.54) | 1.36 (1.31, 1.42) |
| Mar 2021 - Jun 2021 | 1.25 (1.05, 1.49) | 1.65 (1.34, 2.03) | 1.38 (1.33, 1.44) |
| Jul 2021 - Oct 2021 | 1.32 (1.10, 1.57) | 1.43 (1.14, 1.80) | 1.42 (1.36, 1.48) |
| Nov 2021 - Feb 2022 | 1.38 (1.16, 1.63) | 1.24 (0.97, 1.57) | 1.37 (1.32, 1.43) |
| Mar 2022 - Jun 2022 | 1.42 (1.20, 1.67) | 1.41 (1.13, 1.76) | 1.37 (1.32, 1.43) |
| Jul 2022 - Oct 2022 | 1.36 (1.14, 1.62) | 1.51 (1.21, 1.89) | 1.40 (1.34, 1.45) |
| Nov 2022 - Feb 2023 | 1.38 (1.16, 1.63) | 1.41 (1.13, 1.76) | 1.34 (1.28, 1.39) |
| Mar 2023 - Jun 2023 | 1.17 (0.98, 1.41) | 1.33 (1.05, 1.67) | 1.36 (1.31, 1.41) |
| Jul 2023 - Oct 2023 | 1.21 (1.01, 1.46) | 1.29 (1.02, 1.64) | 1.38 (1.32, 1.43) |
| Nov 2023 - Dec 2023 | 1.22 (0.95, 1.57) | 1.17 (0.83, 1.65) | 1.35 (1.28, 1.43) |

# Table S10 Adjusted rate ratios for heart failure incidence for people with each of schizophrenia, bipolar disorder and depression versus those without any of these disorders, for each four-month interval, for all participants, based on a quasi-Poisson model including age, sex, deprivation as well as mental disorder diagnosis, time period and their interaction

| Time period | Schizophrenia | Bipolar disorder | Depression |
| --- | --- | --- | --- |
| Nov 2019 - Feb 2020 | 2.00 (1.81, 2.22) | 1.80 (1.58, 2.05) | 1.43 (1.40, 1.47) |
| Mar 2020 - Jun 2020 | 2.21 (1.97, 2.48) | 2.10 (1.83, 2.42) | 1.47 (1.43, 1.51) |
| Jul 2020 - Oct 2020 | 2.13 (1.91, 2.38) | 2.05 (1.79, 2.34) | 1.53 (1.49, 1.57)* |
| Nov 2020 - Feb 2021 | 2.09 (1.88, 2.32) | 1.85 (1.62, 2.12) | 1.45 (1.42, 1.49) |
| Mar 2021 - Jun 2021 | 2.08 (1.88, 2.30) | 1.80 (1.58, 2.05) | 1.47 (1.44, 1.51) |
| Jul 2021 - Oct 2021 | 2.26 (2.04, 2.50) | 1.78 (1.56, 2.03) | 1.50 (1.46, 1.53)* |
| Nov 2021 - Feb 2022 | 2.08 (1.88, 2.31) | 1.74 (1.53, 1.98) | 1.43 (1.40, 1.47) |
| Mar 2022 - Jun 2022 | 2.00 (1.81, 2.21) | 1.82 (1.61, 2.06) | 1.43 (1.39, 1.46) |
| Jul 2022 - Oct 2022 | 2.11 (1.90, 2.34) | 1.91 (1.68, 2.17) | 1.45 (1.42, 1.49) |
| Nov 2022 - Feb 2023 | 1.96 (1.77, 2.16) | 1.85 (1.64, 2.09) | 1.42 (1.39, 1.46) |
| Mar 2023 - Jun 2023 | 1.98 (1.79, 2.18) | 1.68 (1.48, 1.90) | 1.41 (1.38, 1.44) |
| Jul 2023 - Oct 2023 | 2.07 (1.87, 2.29) | 1.81 (1.60, 2.05) | 1.48 (1.45, 1.52)* |
| Nov 2023 - Dec 2023 | 2.03 (1.76, 2.34) | 1.88 (1.59, 2.23) | 1.41 (1.36, 1.45) |

* Indicates a statistically significant difference in the incidence rate between the asterisked period and the pre-pandemic period

# Table S11 Adjusted rate ratios for heart failure incidence for people with each of schizophrenia, bipolar disorder and depression versus those without any of these disorders, for each four-month interval, for women, based on a quasi-Poisson model including age and deprivation as well as mental disorder diagnosis, time period and their interaction

| Time period | Schizophrenia | Bipolar disorder | Depression |
| --- | --- | --- | --- |
| Nov 2019 - Feb 2020 | 1.98 (1.69, 2.32) | 1.95 (1.63, 2.33) | 1.41 (1.36, 1.46) |
| Mar 2020 - Jun 2020 | 2.48 (2.09, 2.94) | 2.24 (1.84, 2.74) | 1.43 (1.37, 1.49) |
| Jul 2020 - Oct 2020 | 2.14 (1.81, 2.53) | 2.16 (1.79, 2.61) | 1.51 (1.45, 1.57) |
| Nov 2020 - Feb 2021 | 2.11 (1.79, 2.50) | 2.04 (1.68, 2.46) | 1.46 (1.40, 1.51) |
| Mar 2021 - Jun 2021 | 2.11 (1.81, 2.46) | 1.95 (1.63, 2.33) | 1.44 (1.40, 1.49) |
| Jul 2021 - Oct 2021 | 2.31 (1.98, 2.70) | 1.84 (1.52, 2.22) | 1.50 (1.45, 1.55) |
| Nov 2021 - Feb 2022 | 2.25 (1.93, 2.63) | 1.83 (1.51, 2.21) | 1.42 (1.37, 1.47) |
| Mar 2022 - Jun 2022 | 2.09 (1.78, 2.44) | 1.95 (1.63, 2.32) | 1.42 (1.37, 1.46) |
| Jul 2022 - Oct 2022 | 2.25 (1.92, 2.63) | 2.05 (1.72, 2.45) | 1.46 (1.41, 1.51) |
| Nov 2022 - Feb 2023 | 1.98 (1.69, 2.32) | 2.08 (1.76, 2.46) | 1.44 (1.39, 1.48) |
| Mar 2023 - Jun 2023 | 2.12 (1.82, 2.47) | 1.94 (1.64, 2.31) | 1.41 (1.37, 1.46) |
| Jul 2023 - Oct 2023 | 2.08 (1.77, 2.45) | 1.99 (1.67, 2.37) | 1.48 (1.43, 1.53) |
| Nov 2023 - Dec 2023 | 2.08 (1.66, 2.61) | 2.18 (1.72, 2.75) | 1.41 (1.34, 1.48) |

# Table S12 Adjusted rate ratios for heart failure incidence for people with each of schizophrenia, bipolar disorder and depression versus those without any of these disorders, for each four-month interval, for men, based on a quasi-Poisson model including age and deprivation as well as mental disorder diagnosis, time period and their interaction

| Time period | Schizophrenia | Bipolar disorder | Depression |
| --- | --- | --- | --- |
| Nov 2019 - Feb 2020 | 1.99 (1.75, 2.26) | 1.63 (1.36, 1.95) | 1.46 (1.41, 1.51) |
| Mar 2020 - Jun 2020 | 1.95 (1.68, 2.26) | 1.97 (1.63, 2.38) | 1.54 (1.49, 1.60)* |
| Jul 2020 - Oct 2020 | 2.09 (1.83, 2.39) | 1.93 (1.61, 2.31) | 1.55 (1.50, 1.61)* |
| Nov 2020 - Feb 2021 | 2.04 (1.78, 2.32) | 1.67 (1.39, 2.02) | 1.47 (1.42, 1.52) |
| Mar 2021 - Jun 2021 | 2.01 (1.77, 2.28) | 1.61 (1.35, 1.93) | 1.50 (1.45, 1.54) |
| Jul 2021 - Oct 2021 | 2.17 (1.92, 2.46) | 1.72 (1.44, 2.06) | 1.49 (1.45, 1.54) |
| Nov 2021 - Feb 2022 | 1.91 (1.68, 2.18) | 1.66 (1.39, 1.98) | 1.46 (1.41, 1.50) |
| Mar 2022 - Jun 2022 | 1.90 (1.67, 2.15) | 1.70 (1.44, 2.01) | 1.44 (1.40, 1.49) |
| Jul 2022 - Oct 2022 | 1.96 (1.73, 2.23) | 1.77 (1.49, 2.10) | 1.45 (1.41, 1.50) |
| Nov 2022 - Feb 2023 | 1.91 (1.69, 2.16) | 1.62 (1.37, 1.92) | 1.42 (1.37, 1.46) |
| Mar 2023 - Jun 2023 | 1.84 (1.62, 2.08) | 1.41 (1.18, 1.68) | 1.42 (1.38, 1.46) |
| Jul 2023 - Oct 2023 | 2.03 (1.79, 2.29) | 1.63 (1.37, 1.94) | 1.49 (1.44, 1.54) |
| Nov 2023 - Dec 2023 | 1.96 (1.65, 2.33) | 1.58 (1.24, 2.01) | 1.41 (1.35, 1.47) |

* Indicates a statistically significant difference in the incidence rate between the asterisked period and the pre-pandemic period

# Table S13 Adjusted rate ratios for stroke incidence for people with each of schizophrenia, bipolar disorder and depression versus those without any of these disorders, for each four-month interval, for all participants, based on a quasi-Poisson model including age, sex, deprivation as well as mental disorder diagnosis, time period and their interaction

| Time period | Schizophrenia | Bipolar disorder | Depression |
| --- | --- | --- | --- |
| Nov 2019 - Feb 2020 | 1.70 (1.50, 1.93) | 1.84 (1.60, 2.13) | 1.39 (1.36, 1.43) |
| Mar 2020 - Jun 2020 | 1.97 (1.74, 2.23) | 1.79 (1.54, 2.09) | 1.40 (1.36, 1.44) |
| Jul 2020 - Oct 2020 | 1.93 (1.71, 2.18) | 1.85 (1.60, 2.14) | 1.43 (1.39, 1.47) |
| Nov 2020 - Feb 2021 | 2.08 (1.86, 2.33) | 2.01 (1.75, 2.31) | 1.37 (1.34, 1.41) |
| Mar 2021 - Jun 2021 | 1.88 (1.67, 2.12) | 1.94 (1.70, 2.23) | 1.40 (1.36, 1.43) |
| Jul 2021 - Oct 2021 | 1.87 (1.65, 2.10) | 1.95 (1.70, 2.23) | 1.42 (1.38, 1.46) |
| Nov 2021 - Feb 2022 | 1.97 (1.76, 2.21) | 1.66 (1.43, 1.92) | 1.38 (1.34, 1.42) |
| Mar 2022 - Jun 2022 | 1.71 (1.51, 1.93) | 1.82 (1.58, 2.09) | 1.38 (1.34, 1.41) |
| Jul 2022 - Oct 2022 | 1.69 (1.49, 1.92) | 1.94 (1.69, 2.22) | 1.41 (1.37, 1.45) |
| Nov 2022 - Feb 2023 | 1.88 (1.67, 2.11) | 1.63 (1.41, 1.88) | 1.37 (1.34, 1.41) |
| Mar 2023 - Jun 2023 | 1.76 (1.56, 1.98) | 1.80 (1.57, 2.06) | 1.40 (1.37, 1.44) |
| Jul 2023 - Oct 2023 | 1.76 (1.56, 1.99) | 1.60 (1.38, 1.85) | 1.40 (1.36, 1.44) |
| Nov 2023 - Dec 2023 | 1.60 (1.34, 1.92) | 2.03 (1.69, 2.44) | 1.39 (1.34, 1.44) |

# Table S14 Adjusted rate ratios for stroke incidence for people with each of schizophrenia, bipolar disorder and depression versus those without any of these disorders, for each four-month interval, for women, based on a quasi-Poisson model including age and deprivation as well as mental disorder diagnosis, time period and their interaction

| Time period | Schizophrenia | Bipolar disorder | Depression |
| --- | --- | --- | --- |
| Nov 2019 - Feb 2020 | 1.84 (1.54, 2.19) | 2.03 (1.69, 2.45) | 1.36 (1.31, 1.42) |
| Mar 2020 - Jun 2020 | 2.12 (1.78, 2.53) | 1.61 (1.29, 2.01) | 1.41 (1.35, 1.46) |
| Jul 2020 - Oct 2020 | 2.09 (1.76, 2.48) | 1.75 (1.43, 2.15) | 1.39 (1.34, 1.45) |
| Nov 2020 - Feb 2021 | 2.05 (1.73, 2.42) | 2.23 (1.87, 2.66) | 1.36 (1.31, 1.41) |
| Mar 2021 - Jun 2021 | 2.07 (1.76, 2.45) | 2.06 (1.72, 2.47) | 1.38 (1.33, 1.43) |
| Jul 2021 - Oct 2021 | 1.91 (1.60, 2.28) | 1.99 (1.66, 2.40) | 1.43 (1.38, 1.48) |
| Nov 2021 - Feb 2022 | 1.90 (1.60, 2.26) | 1.68 (1.38, 2.05) | 1.34 (1.29, 1.39) |
| Mar 2022 - Jun 2022 | 1.75 (1.46, 2.10) | 1.97 (1.64, 2.37) | 1.36 (1.31, 1.41) |
| Jul 2022 - Oct 2022 | 1.79 (1.49, 2.14) | 1.91 (1.58, 2.30) | 1.36 (1.31, 1.41) |
| Nov 2022 - Feb 2023 | 1.80 (1.51, 2.15) | 1.60 (1.31, 1.95) | 1.33 (1.29, 1.38) |
| Mar 2023 - Jun 2023 | 1.58 (1.31, 1.91) | 1.96 (1.64, 2.34) | 1.37 (1.32, 1.42) |
| Jul 2023 - Oct 2023 | 1.80 (1.50, 2.16) | 1.67 (1.37, 2.03) | 1.39 (1.34, 1.44) |
| Nov 2023 - Dec 2023 | 1.82 (1.41, 2.34) | 2.00 (1.55, 2.57) | 1.37 (1.30, 1.44) |

# Table S15 Adjusted rate ratios for stroke incidence for people with each of schizophrenia, bipolar disorder and depression versus those without any of these disorders, for each four-month interval, for men, based on a quasi-Poisson model including age and deprivation as well as mental disorder diagnosis, time period and their interaction

| Time period | Schizophrenia | Bipolar disorder | Depression |
| --- | --- | --- | --- |
| Nov 2019 - Feb 2020 | 1.55 (1.31, 1.84) | 1.61 (1.30, 2.00) | 1.43 (1.38, 1.49) |
| Mar 2020 - Jun 2020 | 1.80 (1.53, 2.13) | 2.02 (1.65, 2.48) | 1.39 (1.34, 1.45) |
| Jul 2020 - Oct 2020 | 1.75 (1.49, 2.06) | 1.98 (1.62, 2.41) | 1.49 (1.43, 1.55) |
| Nov 2020 - Feb 2021 | 2.06 (1.78, 2.39) | 1.74 (1.41, 2.14) | 1.39 (1.34, 1.44) |
| Mar 2021 - Jun 2021 | 1.68 (1.44, 1.97) | 1.82 (1.49, 2.21) | 1.42 (1.37, 1.48) |
| Jul 2021 - Oct 2021 | 1.79 (1.54, 2.09) | 1.91 (1.57, 2.32) | 1.43 (1.37, 1.48) |
| Nov 2021 - Feb 2022 | 1.99 (1.72, 2.30) | 1.63 (1.32, 2.01) | 1.42 (1.37, 1.48) |
| Mar 2022 - Jun 2022 | 1.64 (1.40, 1.92) | 1.64 (1.33, 2.01) | 1.40 (1.35, 1.45) |
| Jul 2022 - Oct 2022 | 1.58 (1.34, 1.86) | 1.99 (1.65, 2.40) | 1.49 (1.43, 1.54) |
| Nov 2022 - Feb 2023 | 1.90 (1.64, 2.20) | 1.66 (1.36, 2.03) | 1.42 (1.37, 1.47) |
| Mar 2023 - Jun 2023 | 1.86 (1.61, 2.16) | 1.62 (1.32, 1.98) | 1.46 (1.40, 1.51) |
| Jul 2023 - Oct 2023 | 1.70 (1.45, 1.98) | 1.52 (1.24, 1.87) | 1.42 (1.37, 1.48) |
| Nov 2023 - Dec 2023 | 1.40 (1.09, 1.78) | 2.08 (1.60, 2.68) | 1.41 (1.33, 1.48) |

# Table S16 P-values from the likelihood ratio tests for interaction between mental disorder and time period

|  | | P-value | | |
| --- | --- | --- | --- | --- |
|  |  | Myocardial infarction | Heart failure | Stroke |
| Models adjusted for age, sex (overall model only), time period and area-based deprivation | Overall | 0.8794 | 0.0102 | 0.3146 |
|  | Women | 0.8416 | 0.7511 | 0.4885 |
|  | Men | 0.9329 | 0.0499 | 0.1685 |
| Models additionally adjusted for ethnicity | Overall | 0.8662 | 0.0001 | 0.2685 |

# Table S17 Adjusted rate ratios for myocardial infarction incidence for people with each of schizophrenia, bipolar disorder and depression versus those without any of these disorders, for each four-month interval, for all participants, based on a quasi-Poisson model including age, sex, deprivation and ethnicity as well as mental disorder diagnosis, time period and their interaction

| Time period | Schizophrenia | Bipolar disorder | Depression |
| --- | --- | --- | --- |
| Nov 2019 - Feb 2020 | 1.36 (1.16, 1.58) | 1.71 (1.45, 2.03) | 1.43 (1.39, 1.48) |
| Mar 2020 - Jun 2020 | 1.56 (1.34, 1.82) | 1.52 (1.25, 1.85) | 1.41 (1.36, 1.46) |
| Jul 2020 - Oct 2020 | 1.51 (1.30, 1.75) | 1.56 (1.30, 1.86) | 1.42 (1.38, 1.47) |
| Nov 2020 - Feb 2021 | 1.46 (1.26, 1.69) | 1.32 (1.09, 1.59) | 1.39 (1.35, 1.43) |
| Mar 2021 - Jun 2021 | 1.43 (1.24, 1.66) | 1.76 (1.50, 2.06) | 1.39 (1.35, 1.43) |
| Jul 2021 - Oct 2021 | 1.51 (1.30, 1.74) | 1.54 (1.29, 1.83) | 1.42 (1.37, 1.46) |
| Nov 2021 - Feb 2022 | 1.63 (1.42, 1.87) | 1.42 (1.19, 1.69) | 1.37 (1.33, 1.42) |
| Mar 2022 - Jun 2022 | 1.55 (1.35, 1.78) | 1.57 (1.32, 1.85) | 1.38 (1.34, 1.43) |
| Jul 2022 - Oct 2022 | 1.55 (1.34, 1.79) | 1.69 (1.43, 2.00) | 1.41 (1.37, 1.46) |
| Nov 2022 - Feb 2023 | 1.50 (1.30, 1.73) | 1.69 (1.44, 1.98) | 1.39 (1.35, 1.44) |
| Mar 2023 - Jun 2023 | 1.37 (1.18, 1.60) | 1.55 (1.30, 1.83) | 1.39 (1.35, 1.43) |
| Jul 2023 - Oct 2023 | 1.36 (1.16, 1.59) | 1.55 (1.31, 1.85) | 1.42 (1.37, 1.46) |
| Nov 2023 - Dec 2023 | 1.30 (1.05, 1.61) | 1.38 (1.07, 1.77) | 1.39 (1.33, 1.45) |

# Table S18 Adjusted rate ratios for heart failure incidence for people with each of schizophrenia, bipolar disorder and depression versus those without any of these disorders, for each four-month interval, for all participants, based on a quasi-Poisson model including age, sex, deprivation and ethnicity as well as mental disorder diagnosis, time period and their interaction

| Time period | Schizophrenia | Bipolar disorder | Depression |
| --- | --- | --- | --- |
| Nov 2019 - Feb 2020 | 1.99 (1.82, 2.19) | 1.77 (1.57, 1.99) | 1.41 (1.38, 1.44) |
| Mar 2020 - Jun 2020 | 2.20 (1.98, 2.44) | 2.06 (1.82, 2.34) | 1.44 (1.41, 1.48) |
| Jul 2020 - Oct 2020 | 2.12 (1.92, 2.34) | 2.01 (1.78, 2.26) | 1.50 (1.46, 1.53)* |
| Nov 2020 - Feb 2021 | 2.07 (1.88, 2.28) | 1.81 (1.61, 2.05) | 1.42 (1.39, 1.46) |
| Mar 2021 - Jun 2021 | 2.06 (1.88, 2.26) | 1.76 (1.57, 1.98) | 1.45 (1.42, 1.48) |
| Jul 2021 - Oct 2021 | 2.24 (2.04, 2.45) | 1.74 (1.54, 1.96) | 1.47 (1.44, 1.50)* |
| Nov 2021 - Feb 2022 | 2.07 (1.88, 2.26) | 1.70 (1.51, 1.91) | 1.40 (1.37, 1.44) |
| Mar 2022 - Jun 2022 | 1.98 (1.81, 2.17) | 1.78 (1.59, 1.99) | 1.40 (1.37, 1.43) |
| Jul 2022 - Oct 2022 | 2.08 (1.90, 2.28) | 1.86 (1.66, 2.08) | 1.42 (1.39, 1.45) |
| Nov 2022 - Feb 2023 | 1.94 (1.77, 2.12) | 1.81 (1.62, 2.01) | 1.39 (1.36, 1.42) |
| Mar 2023 - Jun 2023 | 1.96 (1.79, 2.14) | 1.63 (1.46, 1.83) | 1.38 (1.35, 1.41) |
| Jul 2023 - Oct 2023 | 2.05 (1.87, 2.24) | 1.76 (1.58, 1.98) | 1.45 (1.42, 1.48) |
| Nov 2023 - Dec 2023 | 2.01 (1.76, 2.28) | 1.83 (1.57, 2.14) | 1.38 (1.34, 1.42) |

* Indicates a statistically significant difference in the incidence rate between the asterisked period and the pre-pandemic period

# Table S19 Adjusted rate ratios for stroke incidence for people with each of schizophrenia, bipolar disorder and depression versus those without any of these disorders, for each four-month interval, for all participants, based on a quasi-Poisson model including age, sex, deprivation and ethnicity as well as mental disorder diagnosis, time period and their interaction

| Time period | Schizophrenia | Bipolar disorder | Depression |
| --- | --- | --- | --- |
| Nov 2019 - Feb 2020 | 1.66 (1.47, 1.88) | 1.83 (1.58, 2.11) | 1.38 (1.34, 1.42) |
| Mar 2020 - Jun 2020 | 1.94 (1.72, 2.19) | 1.77 (1.52, 2.07) | 1.39 (1.35, 1.43) |
| Jul 2020 - Oct 2020 | 1.89 (1.67, 2.13) | 1.82 (1.57, 2.11) | 1.42 (1.38, 1.46) |
| Nov 2020 - Feb 2021 | 2.03 (1.81, 2.27) | 1.98 (1.73, 2.27) | 1.36 (1.33, 1.40) |
| Mar 2021 - Jun 2021 | 1.84 (1.64, 2.07) | 1.92 (1.68, 2.20) | 1.38 (1.34, 1.42) |
| Jul 2021 - Oct 2021 | 1.82 (1.62, 2.05) | 1.92 (1.68, 2.20) | 1.41 (1.37, 1.45) |
| Nov 2021 - Feb 2022 | 1.93 (1.72, 2.17) | 1.64 (1.42, 1.89) | 1.36 (1.33, 1.40) |
| Mar 2022 - Jun 2022 | 1.67 (1.48, 1.89) | 1.79 (1.56, 2.06) | 1.36 (1.32, 1.40) |
| Jul 2022 - Oct 2022 | 1.66 (1.46, 1.87) | 1.91 (1.67, 2.18) | 1.39 (1.36, 1.43) |
| Nov 2022 - Feb 2023 | 1.83 (1.63, 2.06) | 1.60 (1.39, 1.85) | 1.36 (1.32, 1.39) |
| Mar 2023 - Jun 2023 | 1.72 (1.53, 1.94) | 1.77 (1.55, 2.03) | 1.39 (1.35, 1.42) |
| Jul 2023 - Oct 2023 | 1.72 (1.53, 1.94) | 1.57 (1.35, 1.81) | 1.38 (1.35, 1.42) |
| Nov 2023 - Dec 2023 | 1.57 (1.31, 1.88) | 2.00 (1.67, 2.40) | 1.37 (1.32, 1.42) |

# Figure S1 Age-standardised incidence of MI, heart failure and stroke in the general population


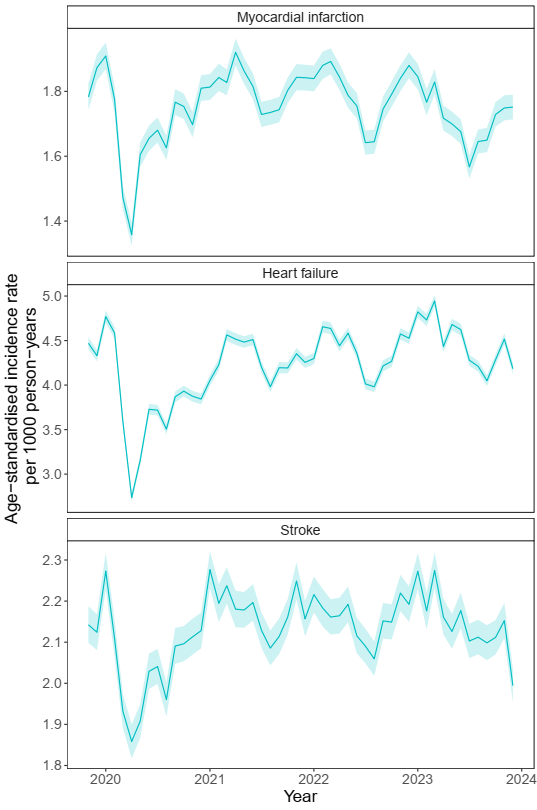


# Figure S2 Age-standardised incidence of MI, heart failure and stroke, by sex and mental disorder diagnosis (amongst people aged 40 to 100 years)


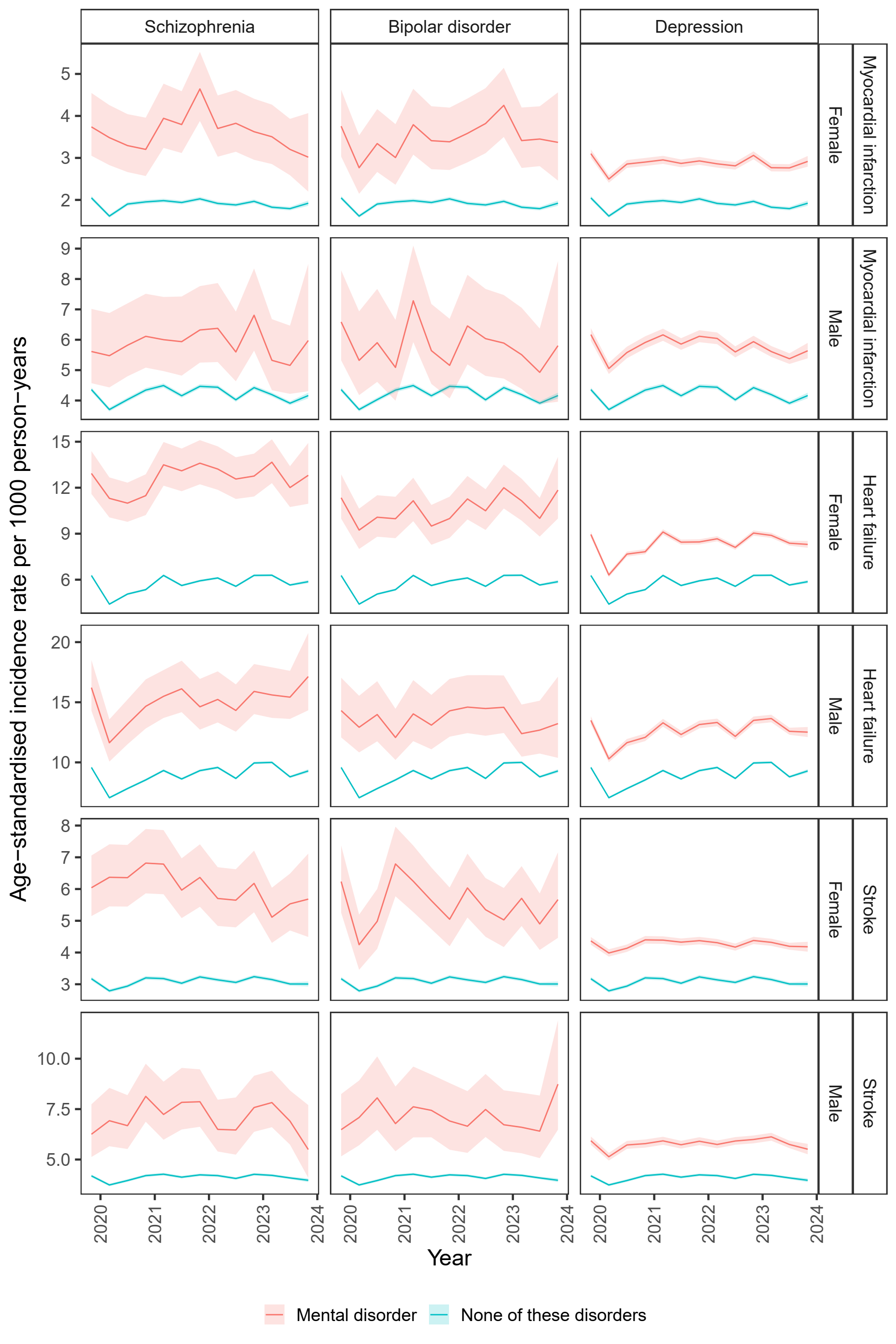


# Figure S3 Rate ratios for MI, heart failure and stroke incidence in those with each of schizophrenia, bipolar disorder and depression versus those without any of these disorders, with additional adjustment for ethnicity (sensitivity analysis)


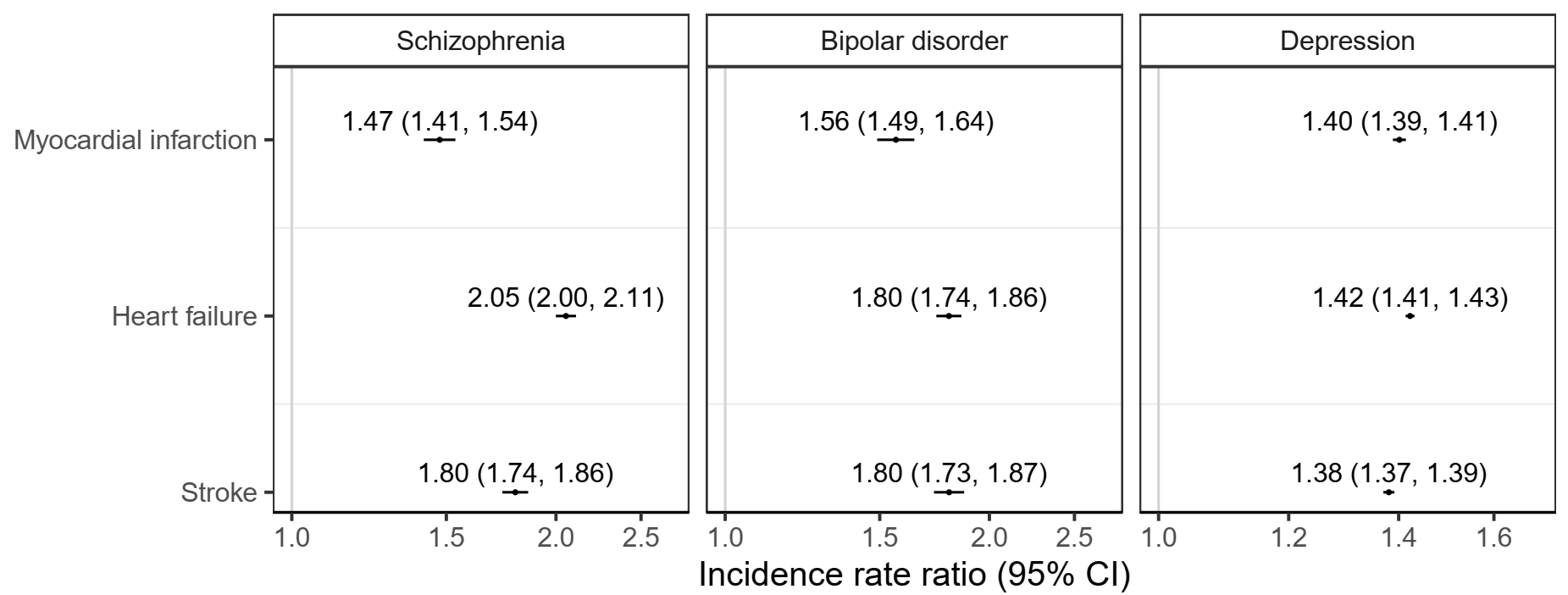

Supplement: online supplemental file 1 [file openhrt-12-2-s001.docx]
